# Lithium systematics in the Krafla volcanic system: comparison between surface rhyolites and felsic cuttings from the Iceland deep drilling project -1 (IDDP-1)

**DOI:** 10.1007/s00410-024-02119-y

**Published:** 2024-04-04

**Authors:** E. A. Cortes-Calderon, B. S. Ellis, T. Magna, L. Tavazzani, P. Ulmer

**Affiliations:** 1 Department of Earth Sciences, Institute of Geochemistry and Petrology, ETH Zürich, Clausiusstrasse 25, 8092 Zurich, Switzerland; 2 Czech Geological Survey, Klárov 3, 11821 Prague 1, Czech Republic

**Keywords:** Lithium, Rhyolite, Post-eruption, Fractional crystallisation, Degassing, IDDP-1

## Abstract

The unexpected discovery of felsic magma by the Iceland Deep Drilling Project-1 (IDDP-1) in the Krafla volcanic system (KVS) presents a unique opportunity to investigate pre-eruptive lithium (Li) dynamics and establish a more direct connection between magma reservoirs and volcanic deposits. Our study provides new insights into Li abundances and isotope compositions in bulk-rock, minerals, and groundmass glass from rhyolitic lavas at KVS, encompassing various stages of groundmass crystallisation. Additionally, we examined felsic cuttings retrieved from the IDDP-1 well, comprising crystal-poor obsidian and crystal-bearing to -rich ‘felsite’ particles. Groundmass glasses from surface lavas show limited variability in K/Na, indicating limited secondary hydration of the glasses and that their Li contents seem to not be affected by this post-eruptive process. Lithium inventories in groundmass glasses and minerals within lavas exhibit variations consistent with the cooling history of the deposit, resembling patterns seen in Snake River Plain ignimbrites. Lithium contents of glassy rhyolitic lavas, whether bulk-rock (avg. 27.2 ± 3.1 μg/g) or groundmass glass (average 28.4 ± 4.7 μg/g), and their bulk isotopic compositions (avg. δ^7^Li =+ 4.4 ± 0.2‰) overlap with those observed in IDDP-1 obsidian cuts (avg. 24.9 μg/g Li in bulk, 28.6 ± 1.5 μg/g in groundmass glass, and δ^7^Li = 4.5 ± 0.2‰). Glassy lavas lacking spherulites may potentially preserve pristine magmatic Li element and isotope compositions, while areas with extensive groundmass crystallisation reveal Li enrichments in phenocrysts. Plagioclases in slowly cooled parts of the deposit record a two-fold increase in Li contents compared to plagioclase found in glassy counterparts, along with evidence of open-system degassing marked by heavier bulk Li isotope compositions and lower bulk Li contents of the crystallised lava portions (avg. δ^7^Li = +7.2 ± 0.1‰ and 7 ± 0.8 μg/g Li) relative to bulk glassy lithologies (avg. δ^7^Li = +4.1 ± 0.1‰ and 28 ± 2 μg/g Li). Partition coefficients derived from IDDP-1 cuts successfully predict Li inventories in vitrophyres of rhyolites on the surface of the KVS. Lithium isotope compositions of the crystal-rich IDDP-1 cuts are significantly heavier (avg. δ^7^Li = +7.2 ± 0.2‰) than lavas and IDDP-1 obsidian cuts, casting doubt on the notion that the IDDP-1 rhyolitic magma could result from the melting of felsite lenses in the KVS. Lithium contents in groundmass glasses within IDDP-1 crystal-rich cuts show higher Li contents (avg. 55.1–60.7 μg/g), correlating with the higher crystal content and an increase in other incompatible elements (avg. 250 μg/g Rb) relative to obsidian cuttings (avg. 75 μg/g Rb).

**Supplementary Information:**

The online version contains supplementary material available at 10.1007/s00410-024-02119-y.

## Introduction

Understanding how lithium (Li) is distributed in the Earth’s crust is critical to improve genetic models of Li-bearing ores (Bowell et al. 2020). Magmatic processes shape the Li elemental and isotopic inventory of the crust by selective transport during fractional crystallisation, partitioning into magmatic fluids, and degassing of melts (Schuessler et al. 2009; Neukampf et al. 2022). Lithium is moderately incompatible in the most common crystallising mineral phases found along typical liquid lines of descent (LLD), enriching felsic derivative liquids in Li (Tomascak et al. 2016; Penniston-Dorland et al. 2017; Schuessler et al. 2009; Iveson et al. 2018; Neukampf et al. 2023). Syn-eruptive degassing removes Li from the melt into exsolved fluid phases inducing kinetic isotope reordering of Li within and among melts, fluids and mineral phases (Vlastélic et al. 2011; Neukampf et al. 2020; Spallanzani et al. 2022).

Post-eruptive modification of Li distribution in volcanic deposits during cooling may, however, obscure pristine magmatic conditions (Zielinski et al. 1977; Ionov and Seitz 2008; Gallagher and Elliott 2009; Ellis et al. 2018; Ellis et al. 2022; Neukampf et al. 2023). While fast-quenched tephras might record near-magmatic Li inventories, the inner portions of lavas and densely welded ignimbrites, which cool slower, might undergo further crystallisation of the groundmass that may enrich phenocrysts in Li by an order of magnitude relative to their rapidly quenched counterparts (Ellis et al. 2018). Where groundmass crystallisation proceeds to near completion, the resulting low proportion of rhyolitic glasses can preserve Li enrichments. At the same time, post-eruptive degassing may also cause loss of Li from the hot deposit as a whole by open-system degassing (Ellis et al. 2018). Lithium inventories of volcanic deposits might be further complicated if secondary hydration of groundmass glass occurs during cooling, removing Li from the glass (Lipman 1965; Ellis et al. 2022). The likelihood of post-eruptive Li mobilisation makes it imperative to identify the latest process that could potentially overprint the intrinsic Li inventories of volcanic deposits before considering processes that happen in magma reservoirs.

Addressing magmatic processes, particularly for rapidly diffusing elements such as Li (Richter et al. 2003; Watson 2017; Holycross et al. 2018), is complicated by the lack of direct access to magma bodies at depth. One fortuitous occasion recently occurred at the Iceland deep drilling project-1 (IDDP-1) well in 2009. This drilling operation, while targeting high-enthalpy supercritical fluids at 4.5 km beneath the Krafla volcanic system (KVS), intersected a rhyolitic magma reservoir at 2.1 km (Pálsson et al. 2014). This unexpected encounter offers an opportunity to assess the pristine magmatic Li inventory of shallow evolved magma reservoirs and to untangle further the observations on Li behaviour in rhyolitic deposits by setting a starting point from where Li inventories are modified by syn-and post-eruptive processes. In this work, we report new Li element contents and isotopic compositions of surface rhyolites in Krafla (Iceland) and explore how Li inventories are related to the cooling history of the deposits. We further characterise the rhyolite cuttings from the magma intersected by the IDDP-1 well in light of Li, discuss the current petrogenetic models proposed for the rhyolitic magma found at depth, and correlate such observations with the lavas at Krafla, all in order to better constrain the behaviour of Li from source to surface. Observations regarding the pathways of Li selective transport in the KVS are compared with the Snake River Plain (SRP), to constrain the effect that post-eruptive processes may exert on rhyolites from different localities.

## Geological setting

**Fig. 1:**
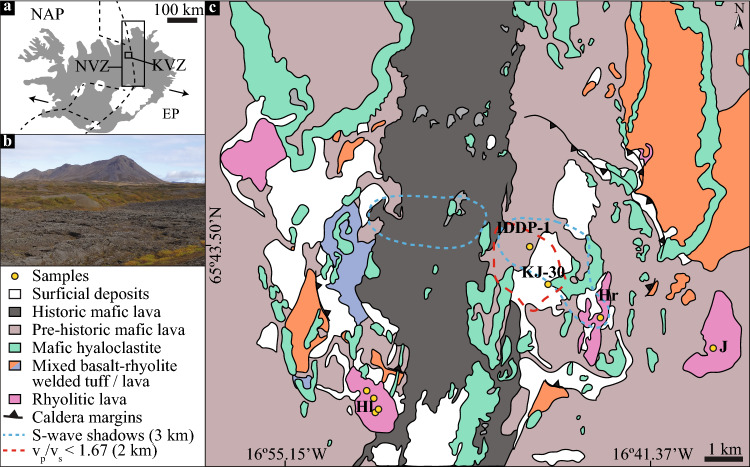
Geological background of the study area. **a** Simplified tectonic map of Iceland after Jóhannesson and Sæmundsson (1998) showing the location of the Krafla volcanic system (KVS) within the northern volcanic zone (NVZ). North American (NAP) and Eurasian (EP) plate boundary shown with dashed lines. Plate motions of ca. 17.5 mm/a indicated by arrows (Drouin et al. 2017). **b** Typical field appearance of the contrasting landforms at Krafla due to the compositional bimodality of volcanic products. Hlíðarfjall (Hl) rhyolitic ridge at the back and historical mafic lava in the front. **c** Simplified geological map of the KVS modified after Sæmundsson et al. (2012), the location of S-wave “shadows” observed during the Krafla Fires (Einarsson 1978) and low-v_p_/v_s_ anomalies (Schuler et al. 2015). Jörundur (J), Hlíðarfjall (Hl) and Hrafntinnuhryggur (Hr) are the rhyolitic domes considered in this study. Iceland Deep Drilling Project-1 (IDDP-1) and Krafla Jotünn-30 (KJ-30) well locations are indicated

Iceland lies atop a divergent plate margin, which separates the North American and Eurasian plates (Fig. 1a), coincident with a melting anomaly, commonly referred to as the Iceland Hotspot (Lawver and Müller 1994). The diversity in volcanic lithologies in Iceland is the result of the interplay between the heterogeneity of the mantle plume beneath Iceland, volcanic eruptions, sea-level changes, fracture zones and the presence or absence of ice sheets during glacial and inter-glacial periods (Bergh and Sigvaldason 1991; Chauvel and Hemond 2000; Fitton et al. 2003; Martin and Sigmarsson 2010; Rooyakkers et al. 2021). Although magmatic activity in Iceland is bimodal in composition, it is mostly dominated by mafic units across time and space (Sæmundsson 1979). Felsic derivatives are primarily confined to central volcanoes located in the vicinity of the plate boundary (Jónasson 2007; Schattel et al. 2014), but there are also off-rift rhyolitic centres (e.g. Snaefelsness Peninsula; Hards et al. 2000).

One locus of rhyolitic volcanism in Iceland is the KVS (Fig. 1), which comprises a ~ 25 km-diameter shield volcano with a central ca. 8 km-wide caldera bisected by a ca. 100 km-long NNE–SSW striking fissure swarm (Fig. 1c; Sæmundsson et al. 2012). The volcanic activity in the area started at least ca. 300 ka and is characterised by eruptions of bimodal composition (Sæmundsson 1991). Rhyolitic volcanism in KVS spans at least 190 ka and is composed of eight main units, an unnamed poorly preserved rhyolitic dome (ca. 190 ka; Sæmundsson and Pringle 2000), the Halarauður ignimbrite related to the central caldera collapse event (ca. 110 ka; Sæmundsson and Pringle 2000; Rooyakkers et al. 2020), three subglacially erupted ridges between 88.7 and 83.3 ka (McGarvie 2009; Hampton et al. 2021), the Hrafntinnuhryggur dike which was emplaced beneath a thin ice layer ca. 24 ka (Sæmundsson and Pringle 2000; Tuffen and Castro 2009), the Hveragil tephra (9000 BP; Sæmundsson 1991) and the Víti tephra as a result of a phreatomagmatic event (1724 CE; Sæmundsson 1991; Montanaro et al. 2021). Rhyolitic lavas form ridge structures that are mostly located around the rim of the central caldera (Fig. 1c), and are proposed to originate by partial melting of altered basaltic crust and/or fractional crystallisation from a more mafic parental magma (Jónasson 1994; Rooyakkers et al. 2021). Although δ^18^O values lower than those of pristine mantle are common in unaltered Icelandic basalts (Burnard and Harrison 2005; Thirlwall et al. 2006), the relatively low δ^18^O values of rhyolites suggest that assimilation of high-temperature altered lithologies is a common process in the petrogenesis of felsic magmas in Iceland (Sigmarsson 1992; Elders et al. 2011; Troch et al. 2020).

As an active geothermal site, the KVS has an extensive set of geophysical surveys and drilling projects aiming to understand the distribution of high-enthalpy geothermal systems. Such studies suggest that the shallower Section (1–2 km) of the KVS consists of subaerial basaltic lavas and hyaloclastites underlain by a basement of mafic intrusives, which show variable extents of alteration in response to the conspicuous hydrothermal system (Scott et al. 2019 and references therein). Low-shear wave velocity zones beneath the KVS imply the potential presence of magma reservoirs between 3 and 5 km depth (Einarsson 1978). However, the presence at shallower depths of rhyolitic magma was confirmed by drilling projects such as the IDDP-1 in 2009 that encountered rhyolitic magma at ca. 2.1 km depth (Pálsson et al. 2014). The origin of the rhyolitic magma has been attributed to either low-degree partial melting of altered basalts (Elders et al. 2011; Zierenberg et al. 2013; Schattel et al. 2014), high-degree partial melting of hypabyssal felsic lenses (Masotta et al. 2018) or Assimilation-Fractional crystallisation of basaltic parental magmas (Rooyakkers et al. 2021). The recovered cuttings from shallower depths, atop the intersected magma batch, comprise hypabyssal felsic particles, commonly referred to as felsites, which host a variable amount of glass (Elders et al. 2011; Saubin et al. 2021). Such glasses have been ascribed to melts that were produced by the re-melting of the host hypabyssal rock after a thermal rejuvenation event (Elders et al. 2011; Zierenberg et al. 2013; Masotta et al. 2018).

**Fig. 2:**
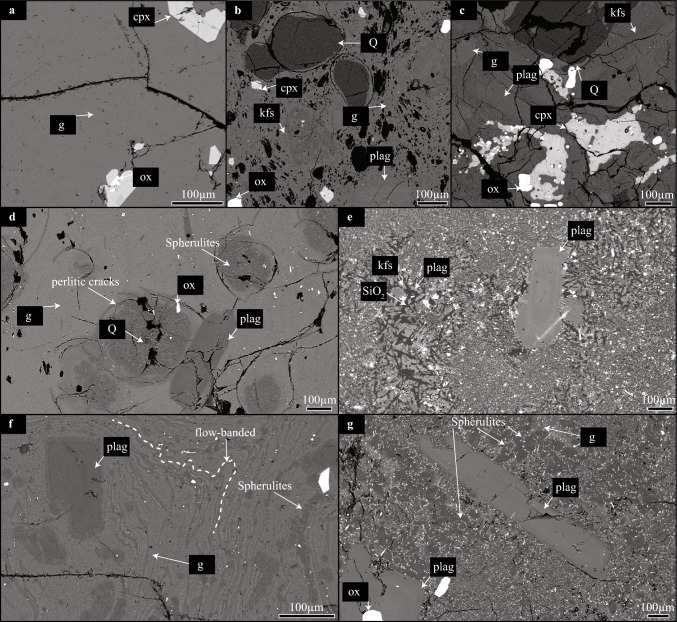
Representative Back-Scattered Electron (BSE) images of the studied samples. Iceland Deep Drilling Project-1 (IDDP-1) cuttings: **a** Obsidian shard, **b** crystal-bearing ‘felsite’ shard with vesiculated glass and **c** crystal-rich ‘felsite’ shard with glass in interstices. Textures of lavas showing different stages of groundmass crystallisation: **d** Spherulite-poor glassy and **e** pervasive groundmass crystallisation textures in Hlíðarfjall lava ridge, **f** spherulite-rich microlite-rich flow-banded glassy and **g** spherulite-bearing glassy textures in Jörundur lava ridge. Abbreviations: groundmass glass (g), clinopyroxene (cpx), Fe-Ti oxide (ox), alkali-feldspar (kfs), plagioclase (plag), quartz (Q), SiO_2_ polymorph (SiO_2_)

Rhyolitic lavas on the KVS are ridges that consist of lobes (Jónasson 1994), varying in groundmass texture from pervasively crystallised inner portions to glassy margins (i.e., vitrophyres). Such lithological variability is typical of relatively thick felsic effusive and explosive deposits and is developed upon cooling of magma after eruption (Lipman 1965; Ewart 1971; Tuffen and Castro 2009; Rowe et al. 2012). The rather complex interplay between cooling rates, nucleation rates and diffusivities of chemical components controls whether a felsic melt is quenched into a glass or crystallised (Swanson 1977; Gardner et al. 2012). Overall, the formation of glass is limited to cooling rates that exceed nucleation rates, but crystallisation of felsic melts is kinetically sluggish, expanding the timeframe where glass is formed due to limitations on chemical diffusivity (Watkins et al. 2009). Fragmentation within the conduit pre-imposes a first-order control on magma cooling. While extensive fragmentation tears magma into clasts that cool fast or can be readily quenched into glass after being erupted (Thomas and Sparks 1992), limited fragmentation promotes coherent magma plugs, or lavas, that cool down irregularly, being capable to quench into glass at their margins and to hold high temperatures at their interiors for protracted periods of time after eruption (Manley 1992). The latter corresponds to the cooling scenario that applies to rhyolitic lavas at KVS. Protracted cooling of a volcanic deposit is, however, not exclusively found in lavas. Densely welded ignimbrites are the result of extensive and rapid agglutination of fragmented magma clasts during or after deposition, keeping the deposit interior hot enough to flow in a lava-like behaviour (Branney and Kokelaar 1992; Ellis et al. 2015). The well-studied Snake River Plain (SRP, USA) rhyolitic ignimbrites fall into such frame, allowing a first-order comparison with the KVS rhyolitic lavas. Crystallisation in the inner portions of rhyolitic lavas, such as at KVS, and densely welded ignimbrites, like at SRP, varies from incipient to pervasive. The interiors of volcanic deposits maintain temperatures above the glass transition, at rather small degree of undercooling, long enough to efficiently crystallise the groundmass (Fig. 2e; Lavallée et al. 2015). Incipient crystallisation is commonly observed by the formation of spherulites (Fig. 2c–f), which might result due to significant undercooling of a melt, representing a transitional zone between vitrophyres and crystalline portions of a volcanic deposit.

In this study, our focus is on three rhyolitic lavas from the KVS: Jörundur, Hlíðarfjall, and Hrafntinnuhryggur, as well as the felsic cuttings recovered by the IDDP-1 well. Our objectives are threefold: to characterise the influence of post-eruptive processes, such as cooling rate, on the inventory of Li in groundmass glass and minerals; to investigate the pristine magmatic Li inventories prior to modification by post-eruptive processes, emphasising the need to understand how these inventories are established within the natural samples, which involves linking Li inventories to crystallisation patterns based on determined partition coefficients; and to evaluate petrogenetic models for the rhyolitic magma intersected by the IDDP-1 well from a Li perspective. By addressing these objectives, we aim to provide insights into the complex processes governing Li distribution in rhyolitic lavas and felsic cuttings, ultimately enhancing our understanding of their petrogenesis.

## Analytical methods

Backscattered electron (BSE) images of mineral phases from lavas and IDDP-1 cuttings were acquired using a JEOL JSM-6390 LA Scanning Electron Microscope (SEM), housed at ETH Zürich, to identify chemical zonation. Cathodoluminescence (CL) images were acquired for IDDP-1 felsite cuttings using a Deben Centaurus CL detector equipped in the SEM. BSE images were used to determine mineral and glass modal compositions of IDDP-1 cuttings using ImageJ software (https://imagej.nih.gov). Major and minor elements in mineral phases and groundmass glass were quantified with a JEOL-JXA 8230 electron probe microanalyser (EPMA), housed at ETH Zürich and equipped with five wavelength dispersive spectrometers (WDS), using an acceleration voltage of 15 kV. Glass shards were measured with 10 nA beam current and 20 µm beam diameter, feldspars at 20 nA and 10 µm, and pyroxenes and Fe–Ti oxides at 20 nA and focused beam. Data were acquired and processed using Probe for EPMA software (Donovan et al. 2021). To test the reproducibility of the measurements during the sessions, analyses were conducted using standard–sample bracketing (Online Resource 1). To minimize the effect of alkali and volatile migration in glasses and feldspars, K and Na were measured at the beginning of the analyses. On-peak measurements were acquired and the analysis time was set according to each material beam sensitivity. Off-peak acquisition was not performed but the background was modelled measuring standards that do not contain the analytes, which is based on a mean atomic number (MAN) background correction (Donovan and Tingle 1996). Time-dependent intensity (TDI) correction was not applied as there were no changes in X-ray intensities as a function of time during the analyses (e.g., alkali migration, Nielsen and Sigurdsson 1981). Quantitative WDS maps from bulk lavas were acquired using the EPMA, which was operated at 15 kV beam acceleration, 100 nA beam current and a focused beam diameter. Pixel size was set to 1 µm by 1 µm and dwell time of 100 ms. Two passes were needed to map eight elements (Si, Al, Ca, Ti, K, Fe, Na, Mg), analysing Na and K in the first pass to reduce the effects of alkali migration. Data for compositional maps were processed using Probe for EPMA, Probe Image and Calc Image software (Donovan et al. 2021). Full acquired dataset and the results of analyses on secondary standards are provided in the Online Resource 1.

Glassy and microcrystalline rhyolitic lava samples were crushed and fused into glass beads with Li_2_B_4_O_7_/LiBO_2_ flux following methodology described in Neukampf et al. (2019) for subsequent bulk-rock analyses (excluding Li). Major element compositions of bulk-rock samples were measured with a PANalytical AXIOS wavelength dispersive x-ray fluorescence (XRF) spectrometer housed at ETH Zürich. Trace element contents of groundmass glass, feldspars and pyroxenes were acquired using a 193-nm Resonetics ArF excimer laser coupled with a Thermo Element XR inductively coupled plasma mass spectrometer (LA-ICP-MS), whereas quartz and fused beads of bulk-rock samples were analysed using an Excimer 193 nm (ArF) GeoLas (Coherent) laser system coupled to a Perkin Elmer Elan 6100 DRC quadrupole ICP-MS, both housed at ETH Zürich. Operational parameters for LA-ICP-MS measurements follow Neukampf et al. (2019) and are detailed in Online Resource 1. Trace element data reduction procedure for quartz and bulk-rock analyses was carried out with the MATLAB-based software SILLS (Guillong et al. 2008), while Iolite4 (Woodhead et al. 2007; Paton et al. 2011) was used for groundmass glass, feldspars and pyroxenes. SiO_2_ content in mineral phases and glass shards obtained by EPMA and CaO content of bulk-rock samples obtained by XRF analyses were used to convert raw counts to absolute contents. To evaluate the accuracy and reproducibility of our instrumental approach, synthetic glasses GSD-1G, BHVO-2G, ATHO-G and GOR128-G were used as secondary reference materials. Trace element data from the studied samples and secondary standards are provided in the Online Resource 1.

Lithium isotope analyses were carried out using a Neptune MC-ICPMS (ThermoFisher Scientific, Bremen, Germany) coupled to an Aridus 2 desolvating unit (Teledyne Cetac, Omaha, USA) at the Czech Geological Survey (Czech Republic). Bulk-rock lava samples and IDDP-1 cuttings were dissolved in a mixture of 27 M HF and 15 M HNO_3_ (6:1 v/v) in closed Teflon vials at 130 °C for 48 h. Solutions were then dried and refluxed repeatedly with small aliquots of 15 M HNO_3_, and the dried residues were finally dissolved in 6 M HCl and equilibrated for 24 h at 80 °C. Lithium purification involved two-stage chromatography following methods detailed elsewhere (Magna et al. 2004, 2006). Lithium contents in purified Li fractions were adjusted to ± 5% of the signal of 10 ppb L-SVEC solution to avoid instrumental bias effects. Conventional standard–sample bracketing was used to obtain Li isotope compositions in unknown samples. The final Li isotope data are reported in the δ notation relative to the NIST RM 8545 reference material (L-SVEC; Flesch et al. 1973) and calculated as δ^7^Li (‰) = [(^7^Li/^6^Li) _sample_ /(^7^Li/^6^Li) _L–SVEC_ − 1] × 1000. The reproducibility of Li isotope measurements was generally better than ± 0.2‰ (two standard deviations, 2 s). The reliability of the analytical and instrumental methodology was verified through the analysis of JR-1 rhyolite (Geological Survey of Japan) and BHVO-2 basalt (US Geological Survey) reference materials; their results are consistent with literature data (Oi et al. 1997; Jochum et al. 2005). Further information regarding the analytical methods and results are provided in the Online Resource 1.

## Results

### Appearance and textures

**Fig. 3:**
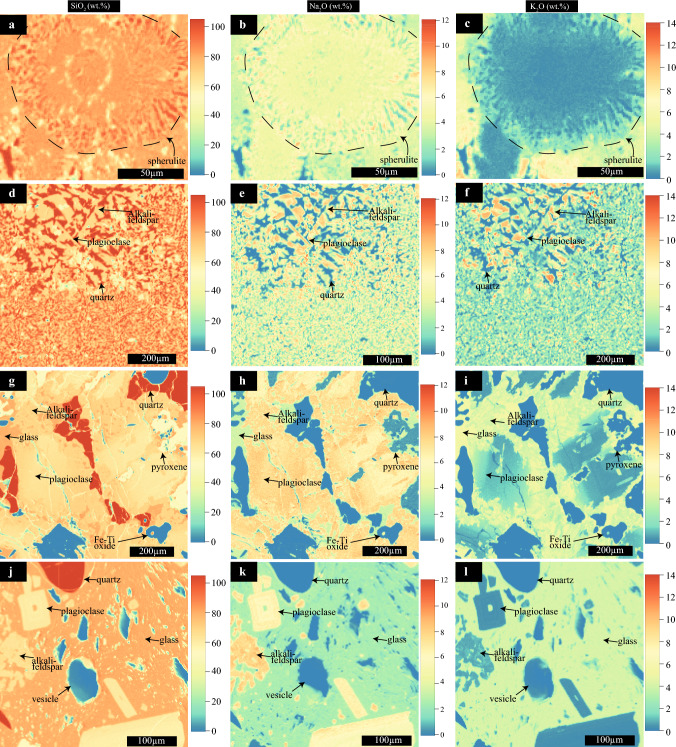
Compositional quantitative maps of SiO_2_ (left column), Na_2_O (central column) and K_2_O (right column) of studied samples obtained with the Electron Microprobe (EPMA) and processed with Probe for EPMA, Calc Image (Donovan et al. 2021) and Surfer software. **a–c** Spherulite-bearing glassy Jörundur lava. **d–f** Hlíðarfjall lava with pervasive groundmass crystallisation. **g–i** crystal-rich shard with glass in the interstices recovered by the Iceland Deep Drilling Project-1 (IDDP-1). **j–l** crystal-bearing shard with vesiculated groundmass glass from IDDP-1

Felsic cuttings recovered from the IDDP-1 well can be classified into three groups: 1) crystal-poor (< 5% vol) obsidian cuttings (Fig. 2a), 2) crystal-bearing (5–50% vol) highly vesiculated glassy cuttings (Fig. 2b), and 3) glass-bearing crystal-rich (> 50% vol) cuttings (Fig. 2c). The latter two groups are commonly referred to in the literature as host rock felsites (Zierenberg et al. 2013; Masotta et al. 2018). While the IDDP-1 crystal-poor obsidian cuttings have a crystal cargo that consists of plagioclase, clinopyroxene and titanomagnetite, the IDDP-1 crystal-rich and crystal-bearing cuttings additionally contain alkali feldspar and quartz. Modal glass proportions of the IDDP-1 cuttings vary from 95 to 13 modal% (void-free normalised). The average modal mineralogy (glass- and void-free normalised) of crystal-bearing cuttings is 42.1% plagioclase, 30.5% alkali feldspar, 22.1% quartz, 3.5% pyroxene 3.5% and 1.8% Fe–Ti oxides, while for crystal-rich cuttings it is 45.7% plagioclase, 26.0% quartz, 15.7% alkali feldspar, 8.9% pyroxene and 3.7% Fe–Ti oxides. While plagioclase generally appears as euhedral to subhedral lath-shaped crystals with oscillatory zoning, alkali feldspars are anhedral and show clear disequilibrium textures (Figs. 2, 3). Pyroxene crystals in the crystal-bearing and obsidian cuttings are subhedral and show exsolution lamellae, whilst they are anhedral with no evident exsolution in the crystal-rich glassy cuttings (Figs. 2, 3; Online Resource 2). Fe–Ti oxides are titanomagnetite and occur as anhedral grains (Figs. 2, 3). Quartz is subhedral to anhedral and it is characterised by bright-CL rims, which vary from very thin (5–10 µm) in glass-bearing crystal-rich cuttings to 50–100 µm wide in crystal-bearing glassy cuttings (Online Resource 2).

Phenocryst modal proportions of glassy lavas, characterised by plagioclase, pyroxene, Fe–Ti oxides and minor apatite and zircon (Rooyakkers et al. 2021), is similar to IDDP-1 obsidian cuttings. Samples from lavas vary mainly in the groundmass crystallinity. While samples taken from rims of the lavas contain abundant glassy groundmass (Fig. 2d), inner portions of the lava ridges continue crystallising during cooling, which reduces the amount of available glass by producing spherulites (Fig. 2d, f, g; Tuffen and Castro 2009), rounded features composed of anhydrous mineral phases that are rich in the components of the SiO_2_–NaAlSiO_3_O_8_–KAlSi_3_O_8_ system (Fig. 3a–c, Ewart 1971). The degree of spherulitisation of lavas is characterised by changes in the mineral composition of the spherulites. Overall, spherulites at KVS consists of aggregates of plagioclase and SiO_2_ polymorphs (Figs. 2d, 3), and increasing the proportion of spherulites in lavas results in the increased proportions of SiO_2_-polymorphs (Figs. 2e, 3). Alkali feldspar is not present in spherulites from Hlíðarfjall or Jörundur lavas (Fig. 2d–f), which is also a common feature in the Hrafntinnuhryggur dike (Castro et al. 2008) or in lavas from other tectonic settings (Ewart 1971). Hlíðarfjall lava with pervasively crystallised groundmass are the only case where alkali feldspar is present among the studied samples (Figs. 2e, 3i). Fully crystallised Hlíðarfjall lava shows a massive texture with a bimodal groundmass crystal size, which is predominantly fine-grained embedding smaller-sized coarse-grained granophyric areas (Fig. 2e). This texture does not preserve precursor spherulitic textures. The phenocryst cargo of the lavas is dominated by plagioclase crystals that are typically elongated and subhedral with oscillatory zoning (Fig. 2d–f), similar to crystal-poor obsidians recovered by the IDDP-1 well.

### Bulk-rock and groundmass glass chemistry

**Fig. 4:**
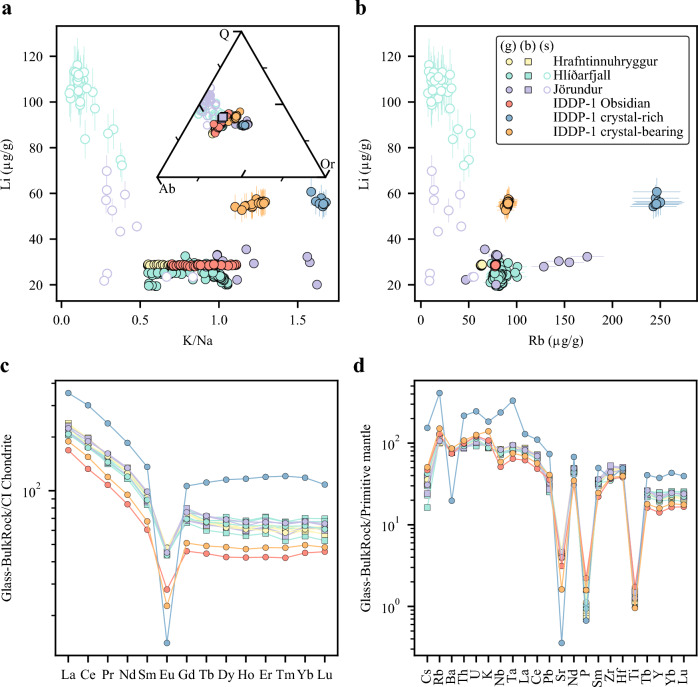
Major and trace element compositions of groundmass glasses (g), spherulites (s) and bulk-rocks (b) obtained by Electron Microprobe (EPMA), X-ray fluorescence (XRF) and laser ablation-inductively coupled plasma-mass spectrometry (LA-ICP-MS). **a** Li and K/Na compositions of groundmass glass and spherulites obtained with LA-ICP-MS. Inset: Major element compositional variations in the SiO_2_ (Q)-NaAlSi_3_O_8_ (Ab)-KAlSi_3_O_8_ (Or) system of groundmass glasses and bulk-rock lavas obtained with EPMA and XRF, respectively. **b** Li versus Rb compositions of groundmass glasses and spherulites obtained with LA-ICP-MS. **c** Rare Earth Elements (REE) diagram normalised to Chondrite-C1 (Sun and McDonough 1989) of bulk-rock lavas and groundmass glasses. Trace elements obtained with LA-ICP-MS **d** Multielement (“spider”) diagram normalised to primitive mantle (McDonough and Sun 1995) of bulk-rock lavas and groundmass glasses. Trace elements obtained with LA-ICP-MS

Bulk lava samples are rhyolitic in composition and show a relatively narrow variation in anhydrous-based SiO_2_, Al_2_O_3_, Na_2_O + K_2_O and Fe_2_O_3(T)_ contents, 74.4–75.8 wt.%, 12.1–12.3 wt.%, 6.8–7.1 wt.%, 3.12–4.19 wt.%, respectively (Online Resource 1). Molar indexes consistently show that bulk lavas have a metaluminous affinity, with Na_2_O + K_2_O/Al_2_O_3_ < 1 and CaO + Na_2_O + K_2_O/Al_2_O_3_ > 1, which concurs with previous studies on KVS rhyolites (Rooyakkers et al. 2021). Groundmass glasses in spherulite-poor (hereafter < 10% vol) lavas are almost identical to major element bulk-rock analyses (Fig. 4a), which is expected from the phenocryst-poor appearance of the samples. Groundmass glasses in spherulite-bearing (heareafter between 10–50% vol) to spherulite-rich (hereafter > 50% vol) lavas show more variation in SiO_2_, K_2_O and Na_2_O and reach greater K_2_O and SiO_2_ contents relative to their respective bulk-rock analyses (Fig. 4a). Spherulites are more enriched in SiO_2_ and Na_2_O and more depleted in K_2_O than the surrounding glass, approaching the SiO_2_–albite join in the SiO_2_–NaAlSi_3_O_8_–KAlSi_3_O_8_ system (Fig. 4a). The presence of significant spherulites (> 50% vol) in lavas is characterised by the least pronounced compositional difference in Na_2_O and K_2_O among spherulites and their respective groundmass glasses but higher difference in SiO_2_ (Fig. 3a-c; Fig. 4a). Spherulites are also characterised by lower Rb contents than their respective groundmass glasses, consistent with previous studies (Fig. 4b; Gardner et al. 2012; Befus et al. 2015). Bulk-rock major element composition of lavas that show considerable amount of spherulitisation are then consistent with groundmass glass major element compositions if spherulite compositions are included. Major element composition of groundmass glass in spherulite-poor lava samples overlaps those from glasses in IDDP-1 obsidian cuttings, while crystal-bearing to crystal-rich cuttings show departures towards higher K_2_O and SiO_2_ contents, similar to groundmass glasses in spherulite-bearing to spherulite-rich lavas (Fig. 4a).

Light rare earth element (LREE) patterns are steep (La_N_/Sm_N_ ~ 2.3–2.8) whereas heavy REE (HREE) patterns are almost flat (Tb_N_/Lu_N_ ~ 1.0–1.1), which is typical for evolved melts (Fig. 4c). The IDDP-1 obsidian with the lowest ΣLREE of 189 µg/g is the least evolved among the IDDP-1 felsic glass shards. Enrichment in LREE in glasses from IDDP-1 felsic cuttings increases with crystal content, reaching ΣLREE of 417 µg/g in crystal-rich cuttings, which indicates limited crystal–melt separation. Negative Eu anomalies of glasses (Fig. 4c) reflect crystallisation of feldspar which is a ubiquitous phase in lavas and cuttings (Fig. 2). While strong depletions in Sr, P and Ti (Fig. 4d) are present in all samples and suggest crystallisation of plagioclase, apatite and Fe–Ti oxides, the crystal-rich IDDP-1 felsic cuttings show the most pronounced anomalies in Ba related to the higher proportion of alkali feldspar crystallisation. The extent of depletions in Sr, P and Ti is more pronounced in the glasses from the crystal-rich IDDP-1 felsic shards, attesting to their more evolved character among the studied glasses (Fig. 4b, d; Zierenberg et al. 2013). In general, the observed succession of trace element enrichment evolves from the groundmass glasses from the crystal-poor IDDP-1 obsidian cuttings to the glass in crystal-bearing IDDP-1 cuttings, followed by all glasses from the lavas, and finally the groundmass glass found in crystal-rich cuttings.

Average Li content of groundmass glasses is 30.3 ± 4.7 (2 s) µg/g for Jörundur, 24.2 ± 2.8 µg/g for Hlíðarfjall and 28.6 ± 0.2 µg/g for Hrafntinnuhryggur (Fig. 4a, b). The scatter in Li contents of groundmass glasses appears to not be affected by secondary hydration of the glasses as there is no clear negative correlation of Li contents in groundmass glasses with increasing K/Na ratios (Fig. 4a; Ellis et al. 2022). When the contrast in Na_2_O and K_2_O content among spherulites and glass is the highest, i.e. the lowest degree of groundmass crystallisation (Fig. 4a, b), Li content in the spherulites reaches up to ca. 110 µg/g (Fig. 4a, b). While Li contents in groundmass glasses from lavas overlap with those from the crystal-poor IDDP-1 obsidian (22.3–32.5 µg/g versus 28.5–28.7 µg/g), the crystal-bearing to crystal-rich IDDP-1 fragments have a two-fold increase in Li content (55.1–60.7 µg/g). This increase in Li content appears to be marked by the presence of alkali feldspar, which typically has the lowest Li partition coefficient among major mineral phases (Neukampf et al. 2023). From crystal-bearing to crystal-rich cuttings there is an increase in incompatible elements, but Li content in glass appears to level out (Fig. 4b).

### Mineral chemistry

**Fig. 5:**
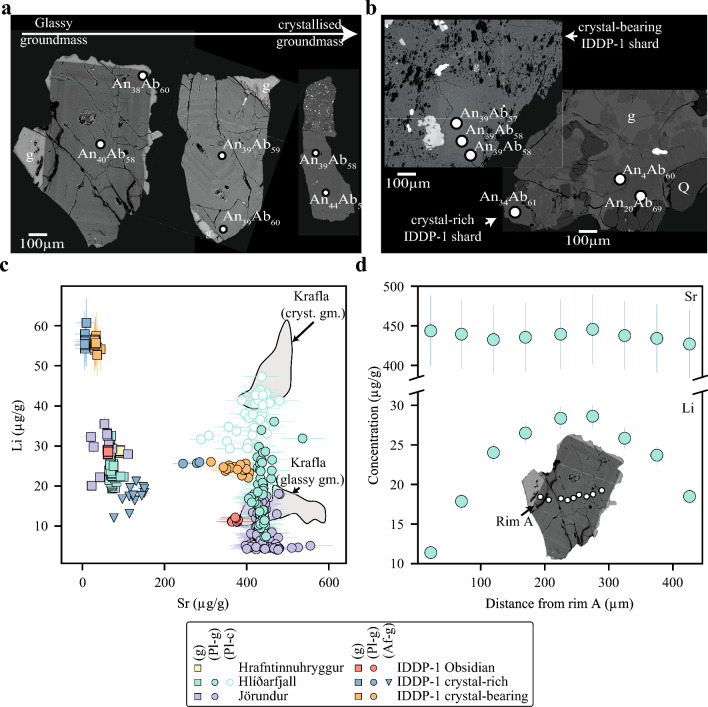
Appearance and compositions of feldspars from lavas and Iceland Deep Drilling Project-1 (IDDP-1) cuttings obtained using Electron Microprobe (EPMA) and laser ablation inductively coupled plasma mass spectrometer (LA-ICP-MS). **a** Representative Back-Scattered Electron (BSE) images of plagioclase in, from left to right, spherulite-poor glassy Hlíðarfjall lava, spherulite-bearing glassy Jörundur lava and Hlíðarfjall lava with pervasive groundmass crystallisation. **b** Representative BSE images of plagioclase in, from left to right, IDDP-1 crystal-bearing and crystal-rich shards. Glass (g), quartz (Q), anorthite (An) and albite (Ab) molar percent contents. Circles represent positions for analyses of major elements with EPMA. **c** Li versus Sr contents obtained with LA-ICP-MS of groundmass glass (g) in lavas and IDDP-1 cuttings, plagioclase in lavas with glassy groundmass (Pl-g), plagioclase in lavas with microcrystalline groundmass (Pl-c) and alkali-feldspar in the IDDP-1 cuttings (Af-g). Krafla plagioclase data from Rooyakkers et al. (2021) added in the background as a function of groundmass texture, crystalline groundmass (cryst. gm.) or glassy groundmass (glassy gm.). **d** Compositional profiles of Li (bottom) and Sr (top) in a representative plagioclase crystal from spherulite-poor glassy Hlíðarfjall lava. Inset shows the distribution of the LA-ICP-MS pits on the BSE image of the corresponding plagioclase grain

Plagioclase is the most abundant phenocryst phase in lavas and IDDP-1 felsic cuttings. As expected, plagioclase phenocrysts in lavas are andesine in composition (An_34-50_Ab_49-64_) regardless of their groundmass texture (Fig. 5a). While plagioclase in IDDP-1 obsidians is similar in composition to that found in lavas, the crystal-bearing IDDP-1 cuttings have feldspars that extend more towards the Ab–KAlSi_3_O_8_ compositional join (An_13-50_Ab_49-70_Or_1-17_ molar; Fig. 5b). Feldspars with higher K contents (orthoclase, Ab_58-66_Or_26-39_ molar) join the crystal cargo when the crystal content increases, which coincides with a liquid line of descent that reaches the quartz–feldspar cotectic from the Na-rich side of the minimum, crystallising more K-rich feldspar with differentiation (Fig. 4a; Tuttle and Bowen 1958). Lithium contents in plagioclases do not vary as a function of plagioclase major elemental composition but groundmass texture (Fig. 5c). Although there is no correlation between Li and anorthite (An) molar content (cf. Coogan 2011) or Sr contents in plagioclase, the ‘uptake’ of Li by alkali feldspars is conspicuously lower than by plagioclase in IDDP-1 crystal-rich cuttings, similar to observations from other felsic centres (Online Resource 2; Neukampf et al. 2023; Lubbers et al. 2022). Rapidly quenched lavas in Krafla, apparent from mostly glassy groundmass, are characterised by plagioclase with quite variable Li contents (7–35 µg/g, Fig. 5c). This variability is not the result of plagioclase grains with different homogeneous Li contents but, instead, it appears to be the result of intra-mineral variations (Fig. 5d). Such variation is characterised by lower Li contents in the rims of the grains relative to their cores, with up to ca. 80% relative depletion, and is unrelated to major or other trace element contents (Fig. 5d). When abundant spherulites are formed in yet glassy lavas (Fig. 2g), plagioclase phenocrysts have the lowest Li contents (4–8 µg/g, Fig. 5c) and no clear intra-mineral variation in Li content. On the contrary, samples with pervasive groundmass crystallisation have typically plagioclases with high and homogenous Li contents (35–45 µg/g). These new findings are strikingly similar to results reported by Rooyakkers et al. (2021) (Fig. 5b).

**Fig. 6:**
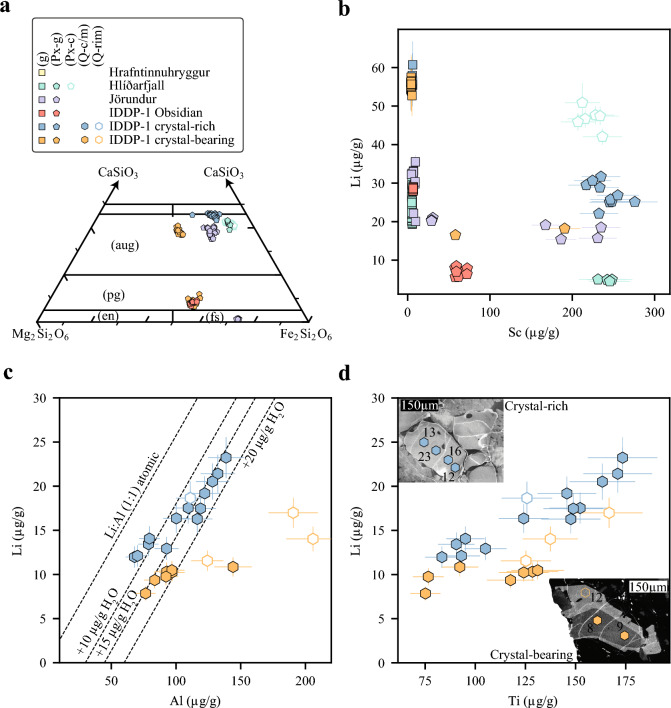
Chemistry of pyroxenes and quartz obtained using Electron Microprobe (EPMA) and laser ablation inductively coupled plasma mass spectrometer (LA-ICP-MS). **a** Major element composition of pyroxenes obtained with EPMA in a section of the CaSiO_3_-Fe_2_Si_2_O_6_-Mg_2_Si_2_O_6_ ternary as a function of groundmass texture, pyroxenes in glassy groundmass (Px-g) or in microcrystalline groundmass (Px-c). Augite (aug), pigeonite (pg), enstatite (en), ferrosilite (fs). **b** Li versus Sc concentrations of groundmass glasses (g), pyroxene in sample with glassy groundmass (Px-g) and pyroxene in sample with microcrystalline groundmass (Px-c) obtained with LA-ICP-MS. **c** Li versus Al concentrations in quartz from crystal-rich and -bearing IDDP-1 shards obtained with LA-ICP-MS. Filled symbols represent analyses obtained either in the core or mantle of the grain (Q-c/m), while open symbols represent analyses taken in the rim of the grain (Q-rim). The isoatomic relationship between Al and Li (1:1) and its departures towards lower Li contents when H^+^ is considered in charge balancing Al^3+^ defects are shown as dashed lines. **d** Li and Ti contents in quartz from crystal-rich and -bearing IDDP-1 shards obtained with LA-ICP-MS. Filled symbols represent analyses either obtained in the core or mantle of the grain, while open symbols represent analyses taken in the rim of the grain. Insets: representative cathodoluminescence (CL) images of quartz grains for each type of IDDP-1 cut

Pyroxenes from Jörundur and Hlíðarfjall lavas are mostly augites (En_6-18_Wo_35-41_Fs_40-50_) with Mg molar fractions (X_Mg_) ranging 0.11–0.31 (av. 0.21, n = 51; Fig. 6a). Jörundur lava additionally contains orthopyroxene, as has also been observed in previous studies (Rooyakkers et al. 2021). Pyroxenes from IDDP-1 crystal-rich cuttings are augitic in composition similar to lavas, while two pyroxene compositions, augite (En_26-30_Wo_27-39_Fs_29-38_) and pigeonite (En_35-39_Wo_6-9_Fs_50-53_), are observed in IDDP-1 crystal-bearing and obsidian cuttings (Fig. 2a, b; Fig. 6a), concurring with previous mineral chemistry on the IDDP-1 felsic cuttings (Masotta et al. 2018). Lithium content in pyroxenes from Hlíðarfjall lavas varies as a function of groundmass texture and has no clear relation with pyroxene major or trace element compositions (Fig. 6b). While Sc contents in pyroxenes from Jörundur lavas vary as a function of pyroxene composition (i.e. clino- vs orthopyroxene), Li contents are indistinguishable between both pyroxene groups (Fig. 6b). The lowest Li contents in pyroxene from Hlíðarfjall lava (< 5 µg/g) are limited to samples with glassy groundmass, while their maximum contents (ca. 47 µg/g, n = 8) are confined to samples with microcrystalline groundmass. There is no clear distinction between Li abundances in orthopyroxene and augite from Jörundur lava ridge, and their Li contents fall between those obtained in pyroxenes from Hlíðarfjall lava. Lithium abundance in pyroxenes from IDDP-1 crystal-rich cuttings is the highest among the IDDP-1 cuttings (av. 27 µg/g, n = 10) but still lower than that in pyroxenes from Hlíðarfjall lava with crystallised groundmass (~ 45 µg/g, Fig. 6b). In contrast to the IDDP-1 crystal-rich fragments, pyroxenes from obsidian cuttings show the lowest Li contents (< 10 µg/g) similar to pyroxenes from Hlíðarfjall glassy lava samples. Lithium contents of pyroxenes from IDDP-1 crystal-bearing cuttings overlap those in pyroxenes from Jörundur lavas and fall between the other two types of cuttings retrieved from the well (Fig. 6b).

Lithium content of quartz grains (12–23.2 µg/g) from IDDP-1 crystal-rich cuttings varies as a function of Al content (67–138 µg/g) and Ti content (83–173 µg/g). Quartz grains from IDDP-1 crystal-bearing cuttings overlap Ti contents of those from crystal-rich cuttings but with lower Li contents (8–17 µg/g) and reach higher Al contents (76–206 µg/g). The quartz population from IDDP-1 crystal-rich cuttings shows a parallel trend to the iso-atomic Li–Al (1:1) line and Li also correlates with Ti contents. Conspicuously CL-bright quartz rims from the IDDP-1 crystal-bearing cuttings (Online Resource 2) show considerable departures from iso-atomic Li–Al trend towards higher Al contents compared to core analyses and only such rims show a correlation with their Ti contents (Fig. 6c–d). Analyses on rims from IDDP-1 crystal-rich cuttings using LA-ICP-MS were limited as they are narrow (Online Resource 2) compared to the laser pit size (ca. 40 µm).

### Lithium mineral-melt partitioning

Partition coefficients between minerals and melt (K_d_^mineral/melt^) measured from IDDP-1 cuttings show that Li behaves as an incompatible element. Partition coefficients for plagioclase from IDDP-1 obsidian cuttings (0.40 ± 0.03) overlap with those obtained from IDDP-1 crystal-bearing and crystal-rich cuttings. Opposite to plagioclase, K_d_^pyroxene/melt^ from IDDP-1 obsidian cuttings (0.24 ± 0.07) are considerably lower than those from IDDP-1 crystal-rich cuttings (0.49 ± 0.11). Partition coefficients for quartz (0.26 ± 0.14) and alkali feldspar (0.30 ± 0.09) are lower than those found for plagioclase and pyroxene in the IDDP-1 crystal-rich to crystal-bearing cuttings.

### Lithium isotope compositions

**Fig. 7:**
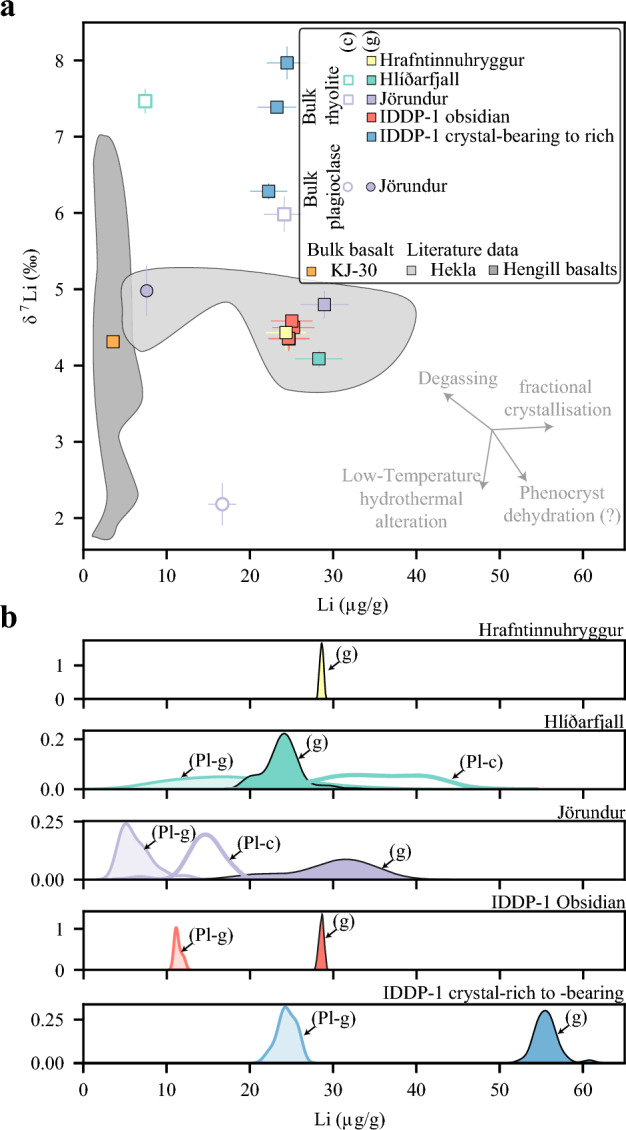
** a** Li element and isotope compositions in bulk samples and bulk plagioclase separates from Krafla using multicollector-inductively coupled plasma-mass spectrometry (MC-ICP-MS). Iceland Deep Drilling Project 1 (IDDP-1) crystal-rich to bearing cuttings are not distinguished by crystallinity, Jörundur (J), Hlíðarfjall (Hl) and Hrafntinnuhryggur (Hr) are the rhyolitic domes and KJ-30 is a Krafla basalt analysed in this study. Li element and isotope compositions of bulk-rock samples from Hekla volcanic system (Schuessler et al. 2009) and basalts from Hengill area (Magna et al. 2011; Verney-Carron et al. 2015) are shown as fields for comparison. **b** Kernel-density distributions of Li inventories in groundmass glasses (g), plagioclases in samples with glassy groundmass (P–g) and plagioclases in samples with crystalline groundmass (P–c) obtained with laser ablation-induced coupled plasma-mass spectrometry (LA-ICP-MS) to compare with bulk Li element compositions obtained with MC-ICP-MS

Bulk samples and plagioclase separates from the KVS are characterised by δ^7^Li values that range from + 2.1 to + 7.8‰ (Fig. 7a). The δ^7^Li values of bulk rhyolitic lavas with glassy groundmass and IDDP-1 obsidian cuttings overlap with the average Li isotope compositions of unaltered MORB (δ^7^Li =  + 3.7 ± 1.0‰, Tomascak et al. 2008), values reported from basaltic to rhyolitic samples from the Hekla volcanic system (3.8–5.0‰; Schuessler et al. 2009) and basaltic glasses from the Hengill area (3.7–6.9‰; Magna et al. 2011). Bulk δ^7^Li compositions from rhyolite lava samples in the KVS vary as a function of groundmass texture. Hlíðarfjall lava samples with pervasive groundmass crystallisation show significant departures towards lower bulk Li content (7.4 ± 0.7 µg/g) and heavier isotopic composition (δ^7^Li =  + 7.47 ± 0.16‰) relative to its spherulite-poor glassy counterpart (28.3 ± 2.8 µg/g and δ^7^Li = 4.09 ± 0.11‰). Lithium-poorer Jörundur lavas show the same trend but with a less pronounced difference (4.8 µg/g and 1.2‰ difference). Although the difference in isotopes between bulk samples is small, analyses of plagioclase separates from Jörundur lavas also show relative departures in Li element and isotope compositions. Plagioclase from spherulite-bearing Jörundur lava is lower in Li abundance (7.6 ± 0.8 µg/g) and isotopically heavier (δ^7^Li = 4.98 ± 0.33‰) than plagioclase from spherulite-rich portions (16.7 ± 1.7 µg/g and δ^7^Li = 2.18 ± 0.28‰). Additionally, the difference in δ^7^Li values between plagioclase and the respective bulk sample is considerably less pronounced in spherulite-bearing Jörundur lavas (0.2‰ difference) than in portions of the lava showing more extensive groundmass crystallisation (3.8‰ difference). The bulk Li element inventories of glassy lavas with poor to moderate spherulitisation obtained using MC-ICP-MS are in good agreement with Li contents of groundmass glass obtained with LA-ICP-MS (Fig. 7b). In the same way, Li element compositions of plagioclases obtained with MC-ICP-MS fall in the range of Li inventories analysed by means of LA-ICP-MS. Bulk Li element compositions of IDDP-1 crystal-bearing to crystal-rich cuttings (23.3 ± 2.2 µg/g) overlap those from bulk analyses on IDDP-1 obsidian cuttings (24.9 ± 2.4 µg/g) but show a higher and more variable range of δ^7^Li values (+ 6.28‰ to + 7.97‰) relative to the IDDP-1 obsidian cuttings (δ^7^Li = 4.45 ± 0.11‰). When comparing the bulk Li element contents of IDDP-1 crystal-rich and crystal-bearing cuttings obtained using MC-ICP-MS with in-situ analyses of minerals and groundmass glass present in such cuttings, the values obtained by bulk analyses are closer to the Li inventories of the crystal cargo than their groundmass glasses. Such an observation hints at the possibility that the fractions analysed using MC-ICP-MS contained mostly crystal-rich fragments.

## Discussion

### The post-eruptive realm

Our results show that post-eruptive Li modifications might not be a limited to the Yellowstone-Snake River Plain (e.g. Ellis et al. 2018), but represent a systematic process in rhyolites. The variation in Li contents of rhyolitic lavas at KVS, the lack of relationship between contents of Li and other trace elements, and the strong association with groundmass textures (Figs. 4a, b, 5c, d,  6b,  7a), collectively imply post eruptive-driven Li mobilisation. Lithium contents of groundmass glass from spherulite-absent to spherulite-poor lavas match those reported from SRP ignimbrites that lack quartz and sanidine phenocrysts (e.g., Greys Landing, Tuff of Wooden Shoe Butte and Tuff of Knob; Ellis et al. 2018; Neukampf et al. 2019). Groundmass glasses from lavas do not show evident secondary hydration by interaction with meteoric water (Fig. 4a) and have a narrow range of Li contents when spherulites are absent or volumetrically unimportant. Although Li has been ascribed to the group of ‘rejected components’ during spherulite crystallisation (Gardner et al. 2012; Befus et al.^2015^), the glass within spherulites at KVS are more enriched in Li than the surrounding glass (Fig. 3b). We propose that such a discrepancy is related to the lack of alkali feldspar in spherulites at KVS, which in turn is a major mineral phase in previously studied spherulites where Li was measured (Gardner et al. 2012; Befus et al. 2015). As Li partitions very weakly into alkali feldspar (Neukampf et al. 2019), this might result in prompting Li rejection from alkali feldspar-bearing spherulites, but this mineral is only found at KVS lavas where the groundmass is pervasively crystallised (Fig. 3a–c). Lithium might then be selectively transported into K-poor spherulites at the expense of H_2_O loss during crystallisation of anhydrous mineral phases. The sequestering of Li into spherulites might induce re-ordering of the Li contents to compensate for the Li-rich glass within spherulite. Although this process might occur relatively rapidly (Watkins et al. 2012), Li is one of the fastest diffusing elements in silicic magmas (Richter et al. 2003; Holycross et al. 2018). Plagioclase from glassy lavas at KVS appears to arrest such Li re-ordering. Lithium profiles in plagioclase from spherulite-poor glassy lavas showing depletions towards the rims of the crystals become relaxed, i.e. homogeneous, when spherulitisation of the lavas increases from spherulite-poor to spherulite-bearing. This might suggest that formation of spherulites requires a temperature–time cooling regime that is similar to the one needed to relax the Li profile in plagioclase. Although we cannot exclude that syn-eruptive degassing could have had a contribution to the amplitude of the arrested concave Li profiles in plagioclase from spherulite-poor glassy Hlíðarfjall lava samples (Fig. 5d), as has been shown for Mesa Falls Tuff plagioclases (Neukampf et al. 2020), its bulk Li isotope composition is not consistent with open-system degassing (Fig. 7a). Vitrophyres that are spherulite-poor to spherulite-bearing in lavas from KVS might potentially record magmatic Li isotopic compositions as there is no evidence of Li mobilisation by secondary hydration, and Li re-ordering due to spherulite crystallisation appears to have occurred under closed-system conditions. When the amount of groundmass crystallisation increases considerably in lavas to either spherulite-rich or pervasively crystallised, such protracted crystallisation may explain the enrichment of Li in plagioclases and their isotopically lighter compositions (Fig. 7a). At this stage, the bulk δ^7^Li values of lavas also start to show signs of post-eruptive open system-degassing, reaching heavier Li isotope compositions and decreasing their bulk Li element contents, similar to what has been observed in the Tuff of Knob (Ellis et al. 2018).

### The pre-eruptive realm

**Fig. 8:**
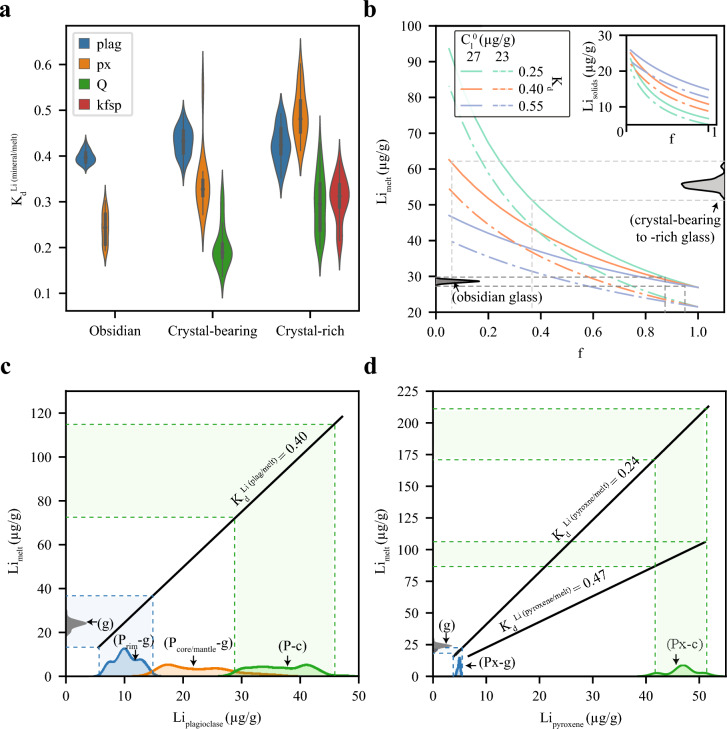
**a** Kernel-density (violin) distribution of apparent partition coefficients of Li (K_d_^Li(mineral/melt)^) for plagioclase (plag), pyroxene (px), quartz (Q) and alkali-feldspar (Kfsp) derived from analyses obtained with LA-ICP-MS of IDDP-1 cuttings. **b** Batch crystallisation model for bulk IDDP-1 obsidian. Two starting compositions (C_l_^0^) were chosen based on average uncertainty of MC-ICP-MS measurements. Three bulk K_d_^Li^ were used based on the range of K_d_^Li(mineral/melt)^ obtained in this study. Kernel-density distribution of Li contents in groundmass glass from IDDP-1 obsidian (left) and crystal-bearing to -rich cuttings (right) are shown on the ordinate. Dashed lines indicate the expected melt content present (f) on the range of Li composition of glasses. Inset: Lithium composition of the bulk solids during batch crystallisation. **c** Predicted Li contents of melts in equilibrium with plagioclases using average K_d_^Li(plagioclase/melt)^ obtained from IDDP-1 obsidians. Kernel-density distribution of Li contents in plagioclases from Hlíðarfjall lavas with glassy (P-g) and microcrystalline groundmass (P–c) are shown on the abscissa. Plagioclases from glassy lava sample are further distinguished between rim (P_rim_-g) and core/mantle (P_core/mantle_-g) analyses. Dashed lines indicate the respective range of Li composition of melt in equilibrium with the plagioclases from Hlíðarfjall lavas. Kernel-density distribution of Li contents in groundmass glass (g) from glassy Hlíðarfjall lava are shown on the ordinate.** d** Predicted Li contents of melts in equilibrium with pyroxenes using average K_d_^Li(pyroxene/melt)^ obtained from IDDP-1 obsidians and average K_d_^Li(pyroxene/melt)^ = 0.47 obtained from IDDP-1 crystal-rich cuttings. Kernel-density distribution of Li contents in pyroxenes from Hlíðarfjall lavas with glassy (Px-g) and microcrystalline groundmass (Px-c) are shown on the abscissa, and Li contents in groundmass glass (g) from glassy Hlíðarfjall lava are shown on the ordinate. Dashed lines indicate the respective range of Li composition of melt in equilibrium with the pyroxenes from Hlíðarfjall lavas

We have shown that Li contents of plagioclase in the KVS do not vary with the An content (Fig. 5c), contrary to experimental study of Coogan (2011) who observed a clear negative trend of Li abundance with An content of plagioclase over the range An ~ 60 to An ~ 90, while our plagioclases are consistently more sodic. Plagioclases from IDDP-1 obsidian cuttings do not record arrested Li profiles suggesting that Li in plagioclase and melt are in equilibrium, regardless of whether the major element compositions of plagioclase and melt might or might not be in equilibrium (Masotta et al. 2018). Our new reported K_d_^plagioclase/melt^ values (Fig. 8a) concur those reported from the Mesa Falls Tuff (Neukampf et al. 2019) but are higher than experimentally derived K_d_^plagioclase/melt^ on rhyodacitic melts (~ 0.23; Iveson et al. 2018). It is important to mention that available partition coefficients derived from experiments of Iveson et al 2018 are fluid saturated while Iceland rhyolites are a relatively dry system. The K_d_^pyroxene/melt^ from IDDP-1 obsidian cuttings is consistent with the reported values from experiments (K_d_^plagioclase/melt^ ~ 0.26; Iveson et al. 2018). The range in K_d_^quartz/melt^ (Fig. 8a) might be the result of variable H_2_O incorporation in quartz among IDDP-1 cuttings (Fig. 6c), with IDDP-1 crystal-bearing cuttings having lower Li contents at the expense of higher H^+^ charge balancing Al defects. Although our reported K_d_^alkali−feldspar/melt^ values (Fig. 8a) are considerably higher than the published apparent K_d_^sanidine/melt^ value of < 0.1 (Neukampf et al. 2023), alkali feldspars in IDDP-1 crystal-rich to crystal-bearing cuttings are anorthoclase rather than sanidine. Plagioclase partition coefficients of Li derived from the IDDP-1 obsidian cuttings show equilibrium relations that match plagioclase rims compositions with groundmass glasses from the spherulite-poor glassy (vitrophyre) portions of Hlíðarfjall lava and plagioclase compositions from pervasive groundmass crystallisation with spherulite compositions (Fig. 8c). Likewise, pyroxene partition coefficients derived from IDDP-1 cuttings show that pyroxene composition from spherulite-poor glassy (vitrophyre) portions of Hlíðarfjall are in equilibrium with groundmass glass compositions (Fig. 8d). Pyroxenes from portions of Hlíðarfjall lava with pervasively crystallised groundmass require a higher partition coefficient to fit spherulite Li compositions, such as those reported in this study from IDDP-1 crystal-rich cuttings. Further analyses on IDDP-1 crystal-rich to crystal-bearing are needed to better understand the change in partition coefficients in pyroxenes compared to IDDP-1 obsidians.

The remarkable lack of volcanic deposits with chemically intermediate compositions in the KVS (referred to as the Daly gap), together with the lower δ^18^O values of felsic products (δ^18^O =  + 1.0–3.2‰; Nicholson et al. 1991) relative to MORB values (δ^18^O ~  + 5.5‰) has challenged the idea that rhyolites are formed by fractional crystallisation. Partial melting of high-temperature altered basalts has been proposed as a mechanism to explain the observed composition of felsic rocks (e.g., Martin and Sigmarsson 2010). The rhyolitic magma encountered by the IDDP-1 well has been proposed to follow the same petrogenetic process (Zierenberg et al. 2013) due to its light O isotope composition (δ^18^O = 3.1 ± 0.06‰; Elders et al. 2011). However, the occurrence of compositional gaps in magmatic suites has been equally plausibly shown to reflect the dynamics of crystal–melt separation within the crust (Dufek and Bachmann 2010; Reubi and Müntener 2022). Additionally, the considerably lower bulk δ^18^O values of altered basalts in Iceland (− 10‰ to − 3.4‰; Hattori and Muehlenbachs 1982) relative to felsic deposits together with new thermal and trace element modelling challenges the partial melting genesis of rhyolites in Iceland (Martin and Sigmarsson 2010; Hampton et al. 2021; Rooyakkers et al. 2021). The “lowness” in δ^18^O of Icelandic rhyolites could have been then inherited by assimilation of altered basalts by basaltic magma coupled with fractional crystallisation (Hampton et al. 2021). We note that fractional crystallisation imparts limited Li isotopic fractionation (Fig. 7a; Schuessler et al. 2009). Lithium isotope compositions of altered basalts in Iceland show that only low-temperature alteration can effectively show departures from MORB values (Fig. 7a; Verney-Carron et al. 2015) and, therefore, ^7^Li/^6^Li ratios are less conclusive regarding whether rhyolites in Iceland are formed by partial melting of altered basalts or assimilation–fractional crystallisation. Masotta et al. (2018) suggested an alternative scenario were the rhyolitic melt intersected by the IDDP-1 well could be generated by high-degree melting of hypabyssal pervasively crystallised felsic lenses such as those retrieved from the IDDP-1 well. Lithium contents of the mineral phases in the IDDP-1 crystal-rich cuttings and its bulk Li isotopic composition are, however, typically lower than Li contents of groundmass glass in IDDP-1 obsidian cuttings. Bulk δ^7^Li analyses on IDDP-1 crystal-rich cuttings are also inconsistent with a high-degree melting scenario as IDDP-1 obsidian cuttings are considerably lighter in Li isotopic compositions, where equilibrium melting, as proposed by Masotta et al. (2018) will favour the heavier isotope (Penniston-Dorland et al. 2017). Although quartz resorption textures have been invoked to support partial melting of hypabyssal felsic lenses, CL images of IDDP-1 crystal-rich and crystal-bearing cuttings (Online Resource 2) consistently show overgrowths of CL-brighter rims with higher Ti and Li contents (Fig. 6d) indicative of crystallisation following resorption (Matthews et al. 2012 and references therein).

The appearance of quartz and anorthoclase in IDDP-1 crystal-bearing to crystal-rich cuttings is in line with higher Li contents in the groundmass glass (Fig. 4b). Comparably to spherulite crystallisation in KVS lavas, where the presence or absence of alkali feldspar controls the Li uptake of spherulites, it appears that KAlSi_3_O_8_ molar composition of the crystallised alkali feldspar strongly influences the extent of Li enrichment of melts. Liquid lines of descent dominated by sanidine fractionation, e.g., evolved alkaline volcanic centres, might then be the most plausible candidates for extreme Li enrichments in derivative melts. However, neither groundmass glasses nor melt inclusions where Li has been measured record extreme Li enrichments in such settings (Neukampf et al. 2023). This might be explained by the buffering or dilution of Li contents in alkaline melts due to recycling of feldspar-dominated cumulates (Sliwinski et al. 2015; Forni et al. 2016; Wolff et al. 2017; Cortes-Calderon et al. 2022; Ellis et al. 2023) during evolution or formation of evolved alkaline derivatives. Batch crystallisation modelling of Li using the bulk Li contents of IDDP-1 cuttings as a starting point can reproduce the measured Li contents in glasses and the observed crystallinities from IDDP-1 cuttings when the range of K_d_^mineral/melt^ values reported here is considered (Fig. 8b).

Equilibrium crystallisation increases Li contents but does not show considerable difference between 60 and 90% crystallinity of the system which is observed in glasses from the IDDP-1 cuttings. Li contents in glasses in the IDDP-1 crystal-rich cuttings might represent a mixture between re-melting of the host-felsite and the IDDP-1 crystal. Melt segregation is then crucial to enrich felsic derivatives in Li. Our dataset indicates that a rhyolitic magma batch, represented by the IDDP-1 obsidian cuttings, stalled in a hypabyssal felsic lens, partly remelted alkali feldspar and quartz followed by crystallisation under equilibrium conditions with the host lithology.

### Bridging magma reservoirs with surface deposits

**Fig. 9:**
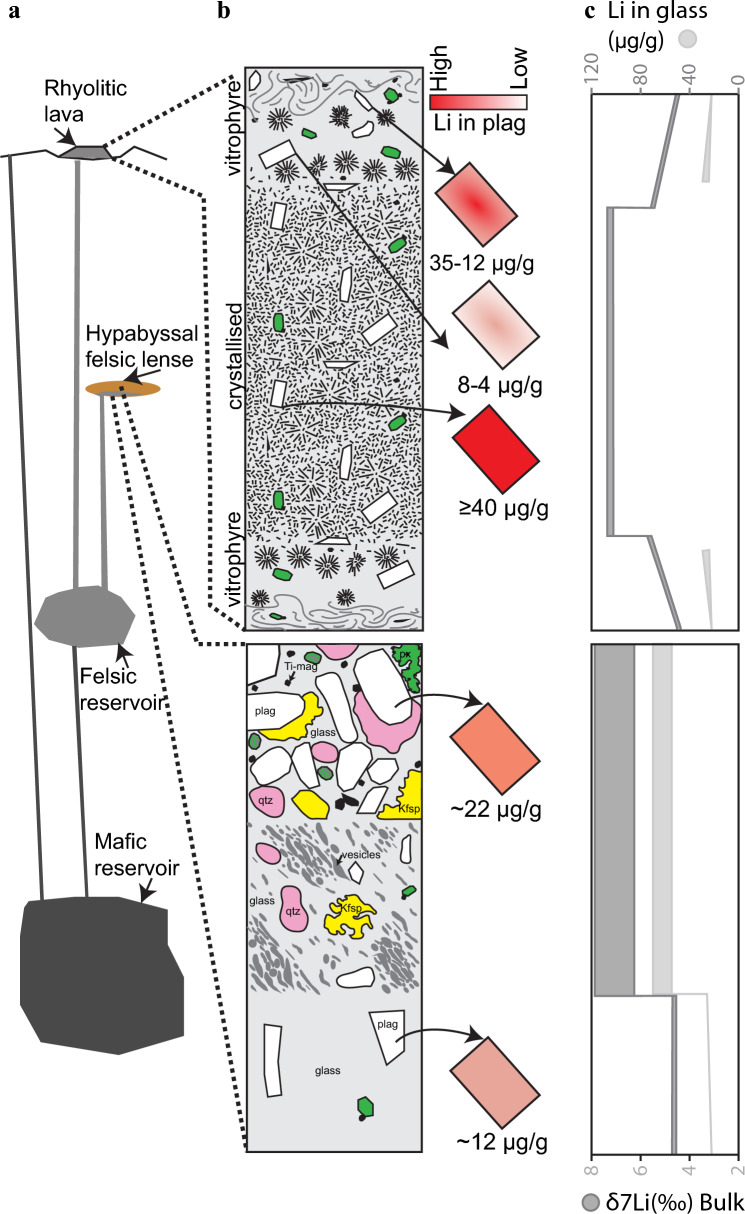
**a** Schematic cross section of the Krafla volcanic system. **b** Schematic generalised stratigraphy of rhyolitic lava domes at Krafla (top) and schematic appearance of the cuttings retrieved from the IDDP-1 well (bottom) showing the Li inventories of plagioclases across the deposits and cuttings. **c** Lithium composition of groundmass glasses and bulk isotope composition across the cuttings and the lava sequences

The overlap in lithium contents and isotopic compositions of IDDP-1 obsidian cuttings and spherulite-poor glassy lavas suggests that rhyolitic batches such as the one encountered by the IDDP-1 well might have risen to the surface, to form the rhyolitic lava ridges present around the Krafla caldera (Fig. 9). This link between the IDDP-1 rhyolite and surface deposits has been shown before with Viti groundmass glasses (Rooyakkers et al. 2020) Plagioclases from the IDDP-1 obsidian cuttings show no changes in Li composition within crystals stressing that such plagioclases were in equilibrium with the surrounding melt, at least from Li perspective, and that partition coefficient derived from such cuttings can be used to understand the distribution of Li in the rhyolitic deposits. Currently, experimental work on Li in felsic derivatives is limited (Neukampf et al. 2019). The IDDP-1 cut can provide similar Li results (e.g. element and isotope partitioning) to what a melting experiment of a rhyolite at dry conditions might retrieve. Whilst it is possible that the magma encountered at depth underwent degassing before the IDDP-1 drilling, our isotope data does not show any significant departures indicative of open-system disequilibrium degassing. Although vesiculation in crystal-bearing cuttings might suggest significant degassing, these vesicles are drilling-induced (Saubin et al. 2021). Nevertheless, there is evidence suggesting that the magma encountered by the well experienced closed-system degassing before rising to its current depth (Elders et al. 2011; Saubin et al. 2021), so it might not represent Li element inventories of deeper felsic shallow magma reservoirs.

Linking Li inventories of rhyolitic deposits with their magma reservoirs require understanding if this inventories might be modified by post-eruptive processes such as differential cooling through the volcanic deposit or secondary hydration by surficial waters. The IDDP-1 cuttings provide the closest and direct assessment to natural magmatic pristine Li compositions of felsic melts ever analysed.

## Conclusions

Our study builds upon the fortuitous discovery of felsic magma by the IDDP-1 in the KVS, providing a unique opportunity to explore pre-eruptive lithium (Li) dynamics. Through analysis of bulk-rock, minerals, and groundmass glass from rhyolitic lavas at KVS, spanning various stages of groundmass crystallisation, we have elucidated crucial insights into how Li abundances and isotope compositions are controlled in volcanic deposits. Notably, our findings corroborate the established understanding that Li can be effectively mobilised during the cooling of rhyolitic lavas, akin to processes observed in fallout deposits and ignimbrites from the Snake River Plain. The degree of groundmass crystallisation emerges as a pivotal factor influencing the Li inventory of mineral phases and glass, with rapidly quenched portions of rhyolitic lava exhibiting plagioclase crystals with consistently depleted Li concentrations in their rims relative to the cores. Furthermore, our analyses indicate that glass within spherulites typically has higher Li concentrations compared to the groundmass glass. In lithologies characterised by a greater proportion of spherulites, plagioclase phenocrysts show minimal core-to-rim variation in Li, suggesting diffusion processes prior to quenching. Conversely, devitrified lavas with pervasive groundmass crystallisation exhibit elevated Li contents in plagioclase and pyroxene crystals compared to rapidly quenched portions, indicative of complex Li reordering mechanisms during crystallisation. The findings underscore the intricate interplay between crystallisation dynamics and Li behaviour in volcanic systems like the KVS. Additionally, our investigation into lithium partition coefficients derived from IDDP-1 cuttings supports a moderate incompatibility of Li within the phenocryst assemblage. Similar partition coefficients observed between plagioclase, pyroxene, and groundmass glass in spherulite-poor glassy portions of surface rhyolitic lavas further validate that glassy lavas, spherulite-poor, might resemble magmatic conditions. Lithium isotope compositions of the crystal-rich IDDP-1 cuts cast doubt on the notion that the IDDP-1 rhyolitic magma could result from the melting of felsite lenses in the KVS.

### Supplementary Information

Below is the link to the electronic supplementary material.

Supplementary file1 (XLSX 809 kb)

Supplementary file2 (PDF 98833 kb)

## Data Availability

All data regarding analyses and sample descriptions are in the supplementary information.
