# Supplementary material for: Lithium systematics in the Krafla volcanic system: comparison between surface rhyolites and felsic cuttings from the Iceland deep drilling project -1 (IDDP-1)
Source: Contrib Mineral Petrol. 2024 Apr 4;179(4):37. doi: 10.1007/s00410-024-02119-y (PMC10995055; doi:10.1007/s00410-024-02119-y)
Supplement: Supplementary file 2 — Supplementary file2 (PDF 98833 kb) [file 410_2024_2119_MOESM2_ESM.pdf]

Online Resource 2: Supplementary figures

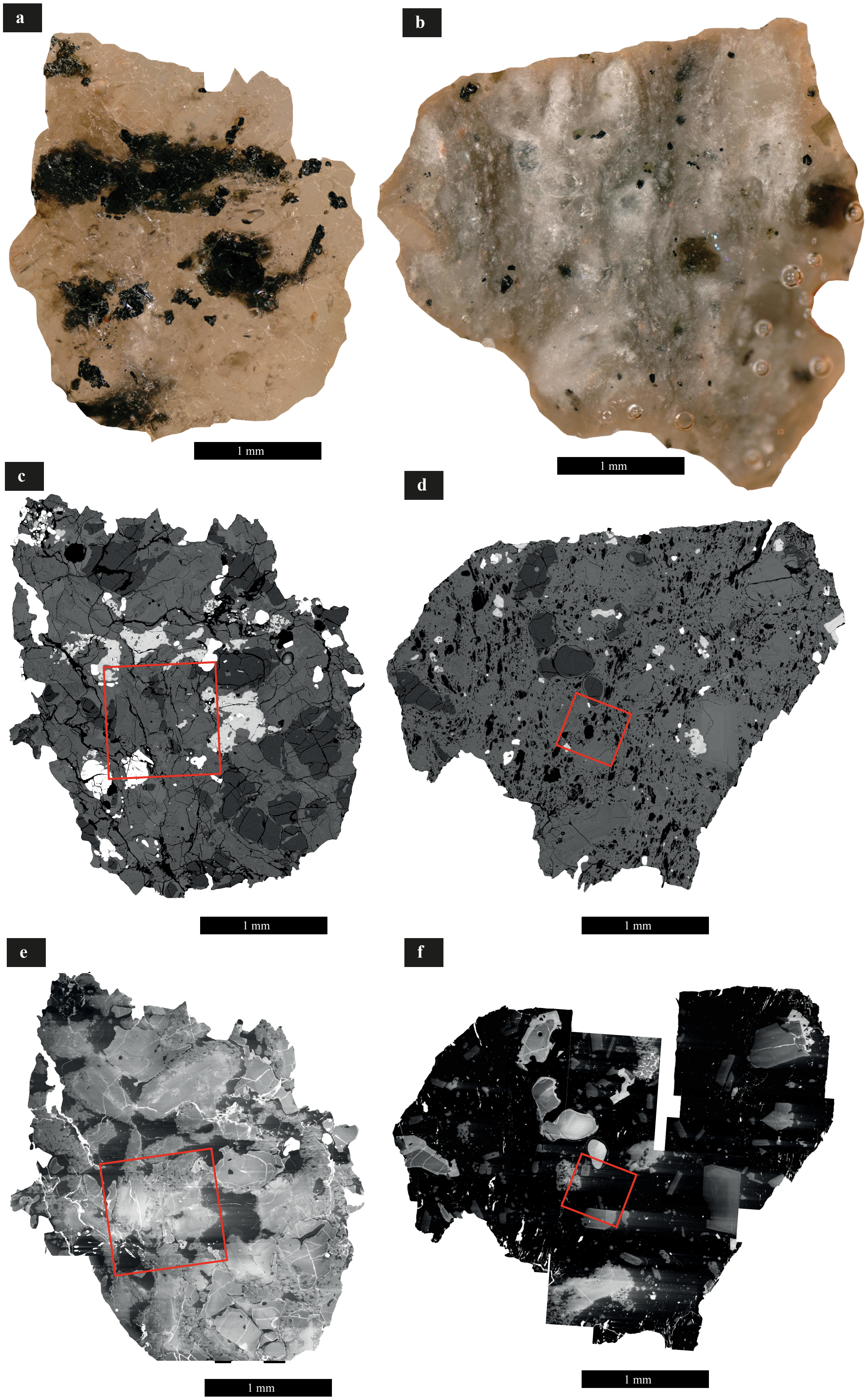

Fig. 1 Appearance of crystal-rich and -bearing rhyolites recovered by the IDDP-1. a-b Transmited light optical images. c-d Back-scattered electron (BSE) images obtained with the Scanning Electron Microscope (SEM). d-e Cathodoluminescence (CL) images obtained with the SEM. Note that quartz grains constantly have brigther CL rims. Red rectangles indicate the approximate areas where the quantitative maps of Fig. 2-3 were acquired.

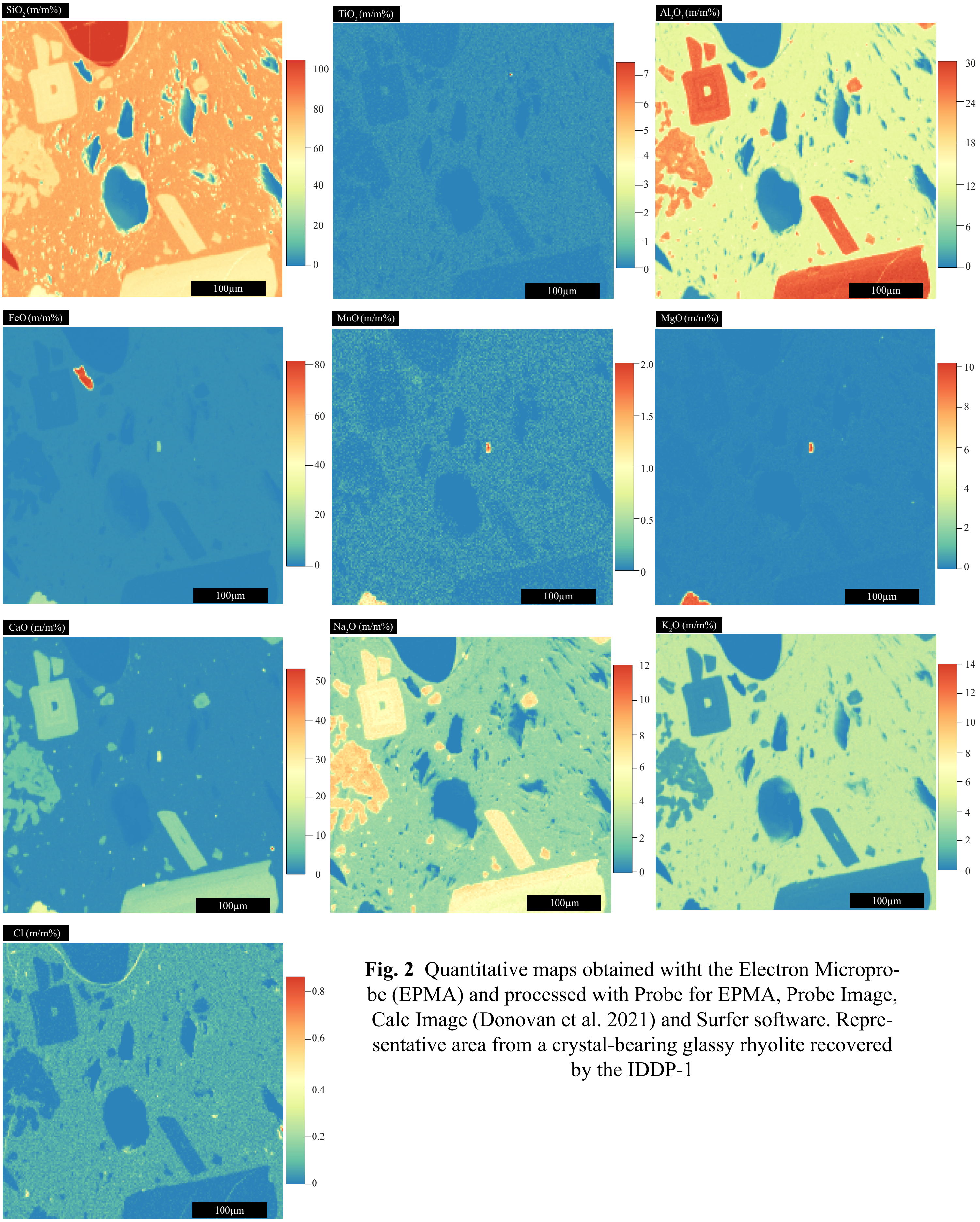

Fig. 2 Quantitative maps obtained witht the Electron Microprobe (EPMA) and processed with Probe for EPMA, Probe Image, Calc Image (Donovan et al. 2021) and Surfer software. Representative area from a crystal-bearing glassy rhyolite recovered by the IDDP-1

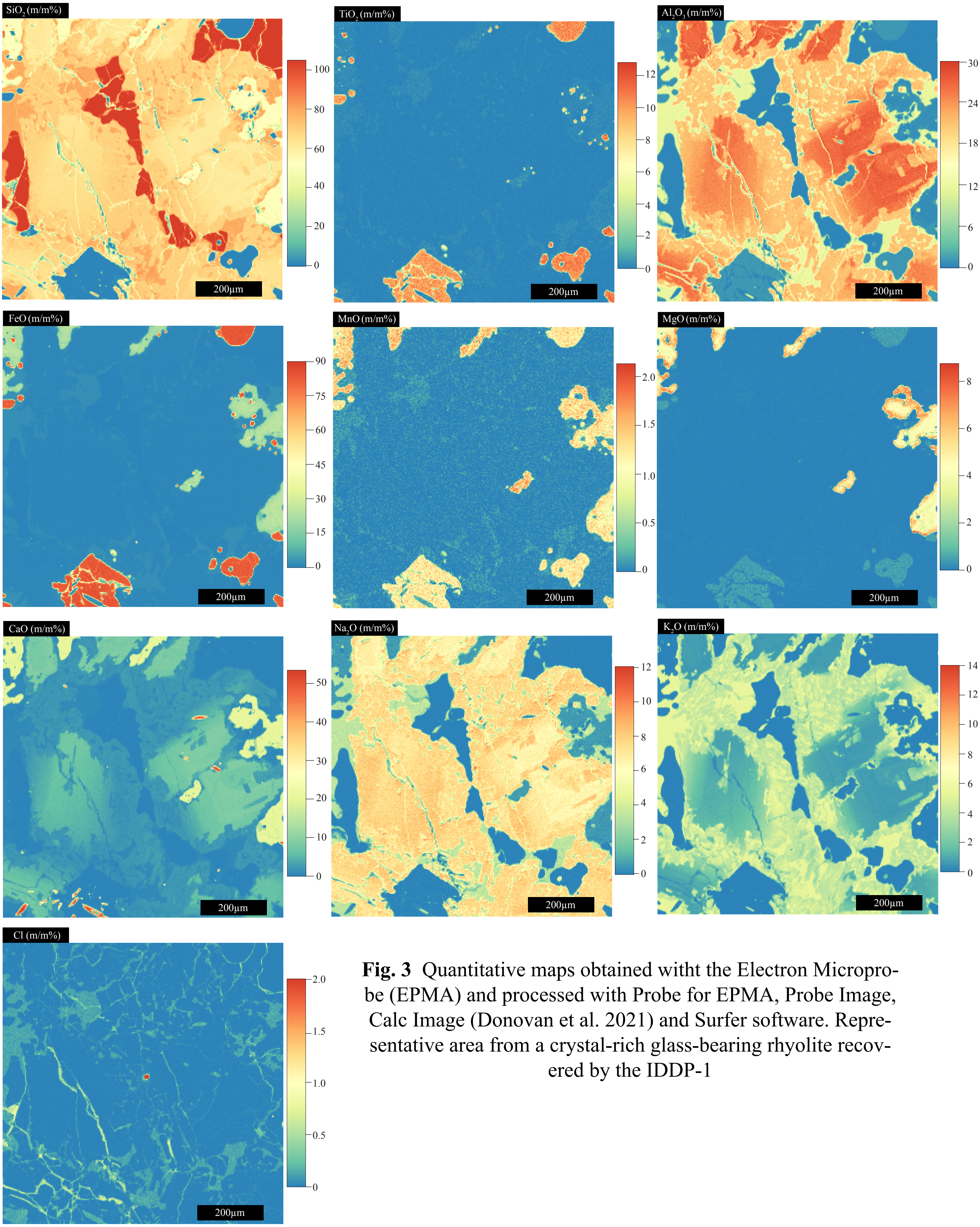

Fig. 3 Quantitative maps obtained witht the Electron Microprobe (EPMA) and processed with Probe for EPMA, Probe Image, Calc Image (Donovan et al. 2021) and Surfer software. Representative area from a crystal-rich glass-bearing rhyolite recovered by the IDDP-1

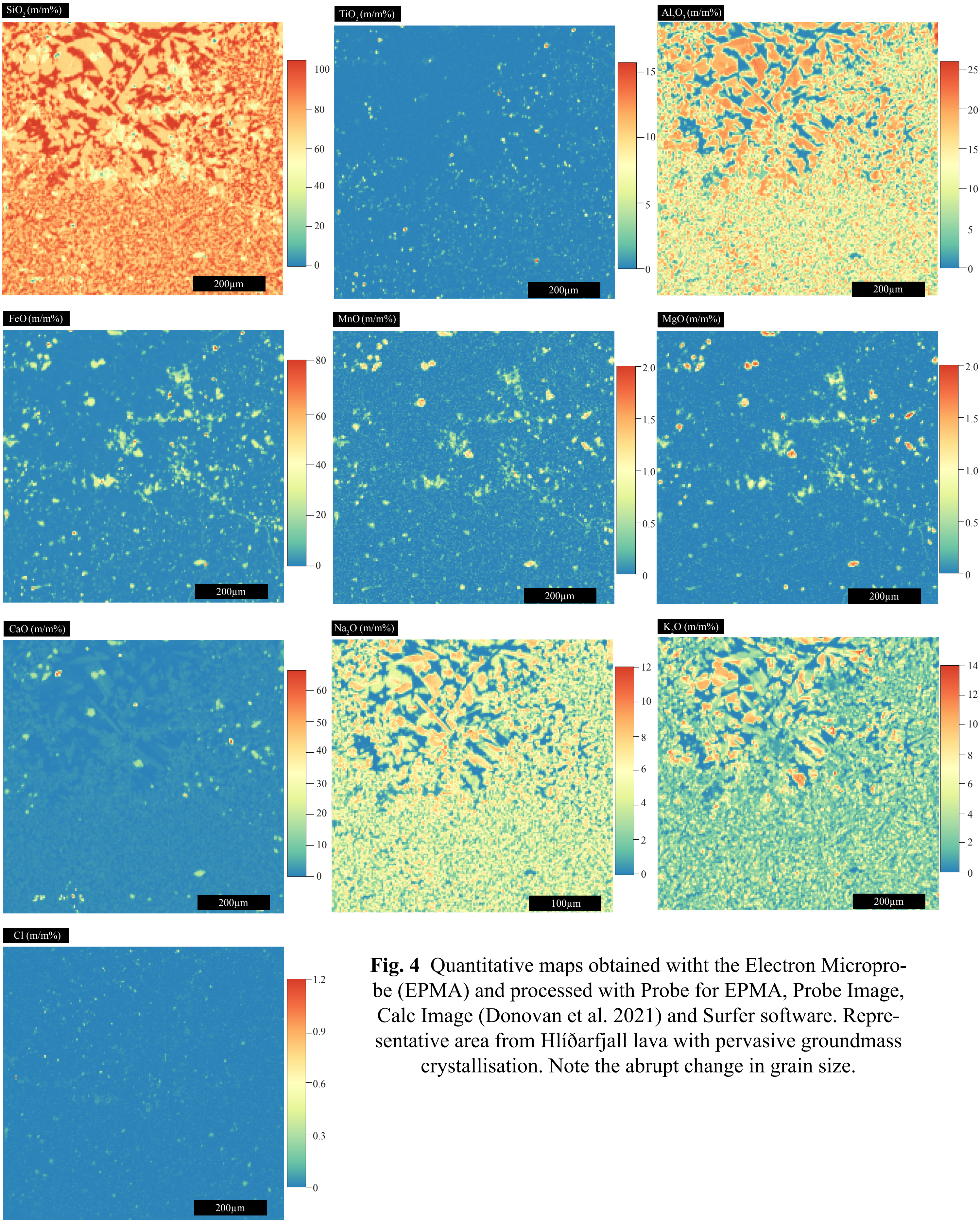

Fig. 4 Quantitative maps obtained witht the Electron Microprobe (EPMA) and processed with Probe for EPMA, Probe Image, Calc Image (Donovan et al. 2021) and Surfer software. Representative area from Hlíðarfjall lava with pervasive groundmass crystallisation. Note the abrupt change in grain size.

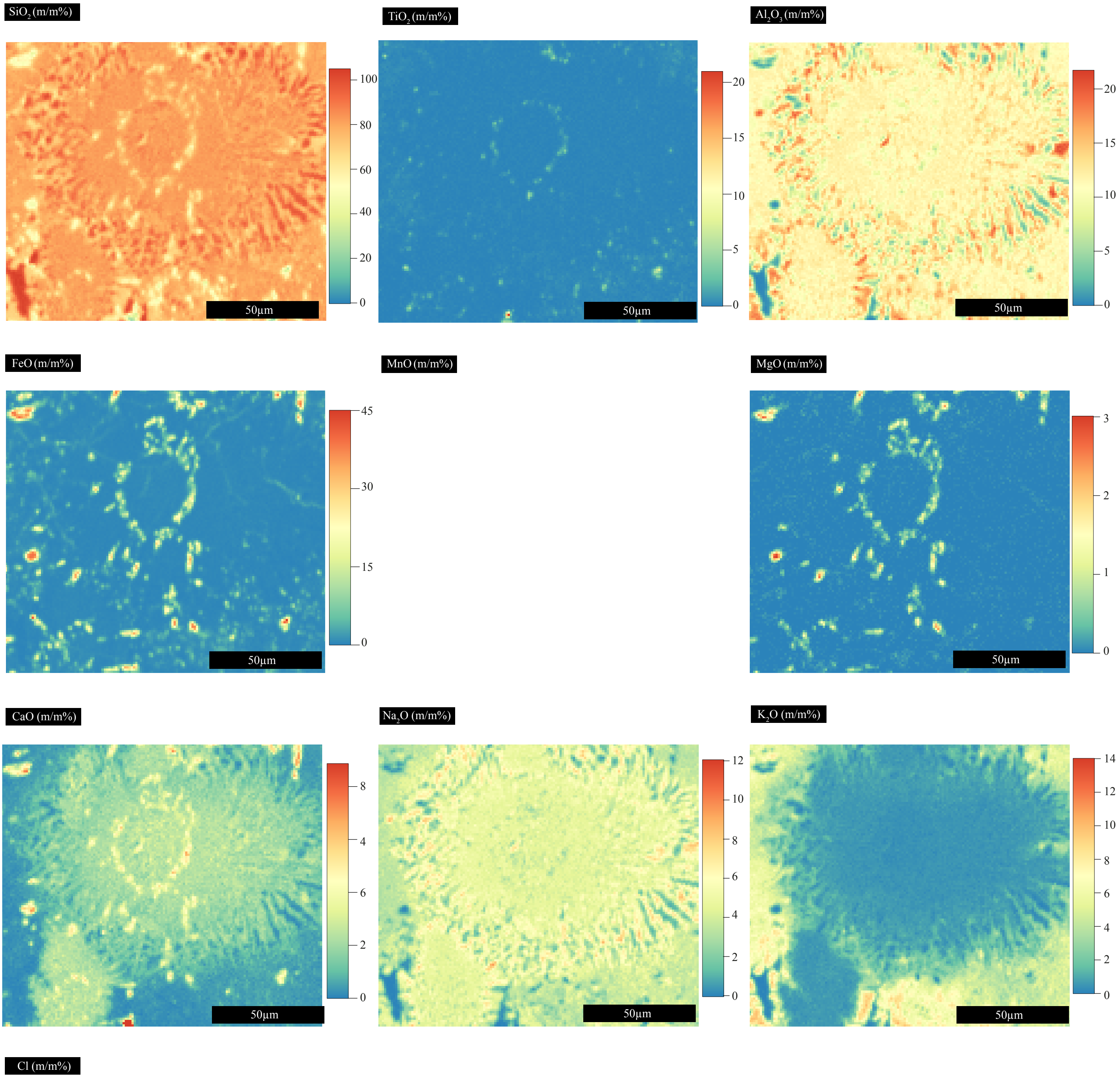

Fig. 5 Quantitative maps obtained witht the Electron Microprobe (EPMA) and processed with Probe for EPMA, Probe Image, Calc Image (Donovan et al. 2021) and Surfer software. Representative area from Jörundur lava with widespread spherulitisation. Cl and MnO were not measured for this sample.

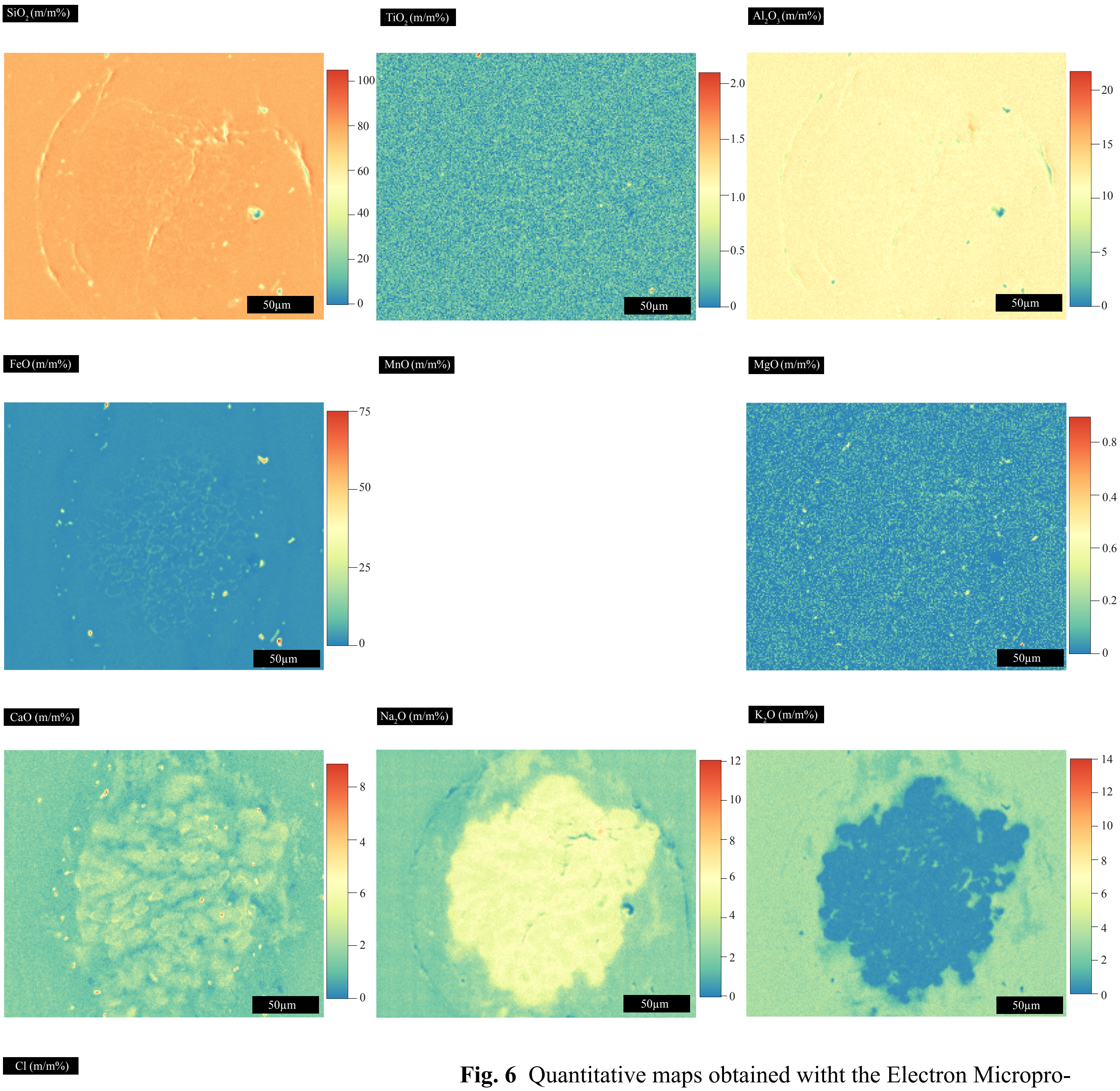

Fig. 6 Quantitative maps obtained witht the Electron Microprobe (EPMA) and processed with Probe for EPMA, Probe Image, Calc Image (Donovan et al. 2021) and Surfer software. Representative area from Hlíðarfjall lava with very incipient groundmass crystallisation. Cl and MnO were not measured for this sample.

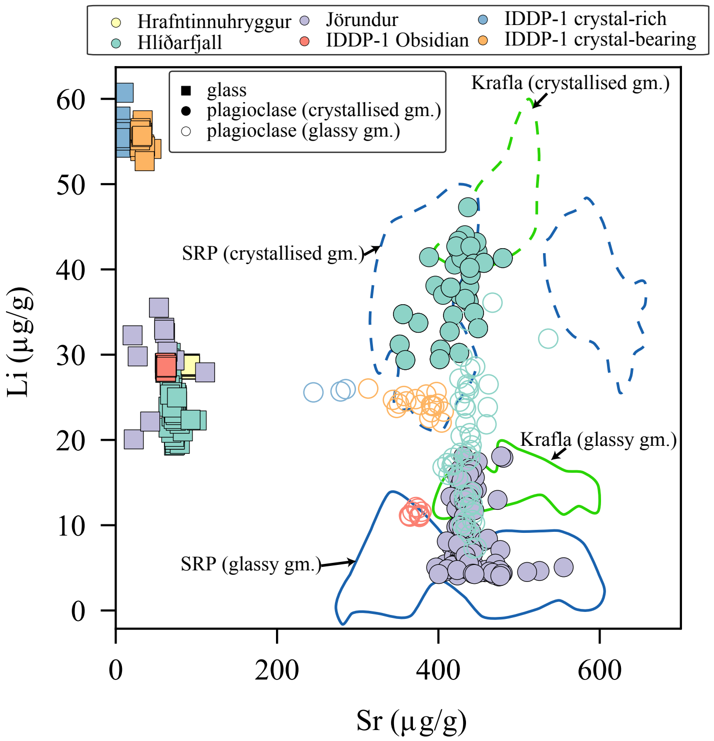

Fig. 7 Li and Sr concentrations in plagioclase and groundmass glass as a function of texture. Snake River Plane (SRP) plagioclase data in the background for comparison from Ellis et al. (2018) and Krafla plagioclase data from Rooyakkers et al. (2021).

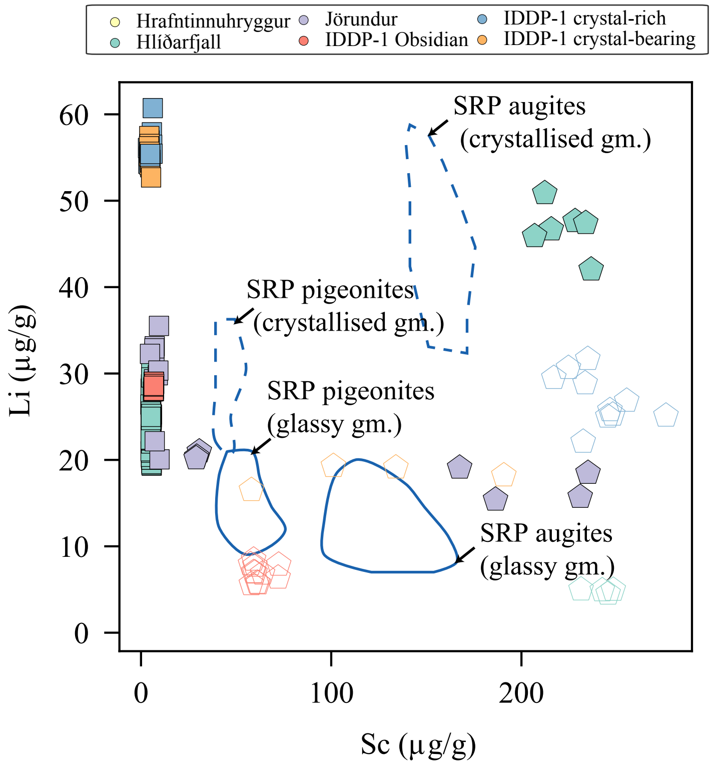

Fig. 8 Li and Sc concentrations in pyroxenes and groundmass glass as a function of texture. Snake River Plane (SRP) pyroxene data in the background for comparison from Ellis et al. (2018).

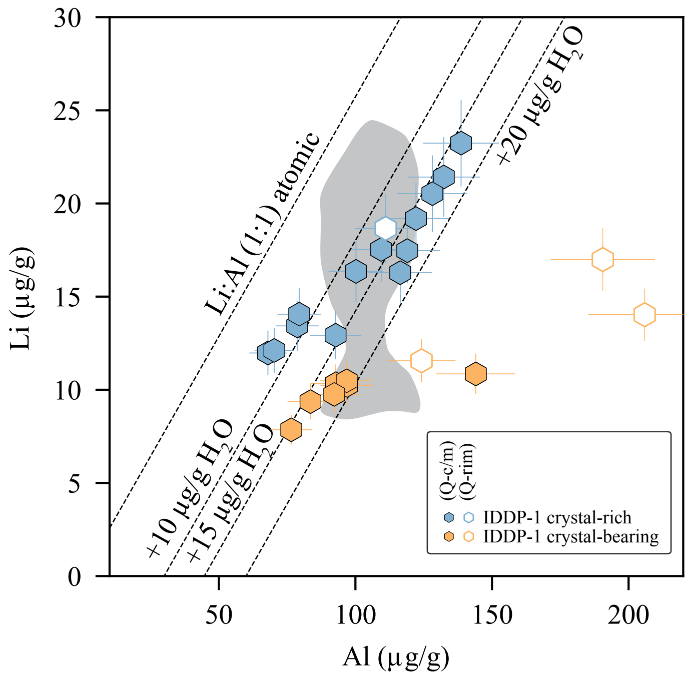

Fig. 9 Li and Al concentrations in quartz and groundmass glass as a function of texture. Filled symbols (Q-c/m) refer to measurement on the mantle or core of the quartz grains, while non-filled ones refer to measurements on the rim (Q-rim). Isoatomic relationship between Al and Li and its departures towards lower Li contents when H^+ is also considered in charge balancing Al^{3+}defects in quartz. is shown in dashed linesMesa Fall Tuff (MFT) quartz data in the background for comparison from Neukampf. (2022)
